# HIV-1 Polymerase Inhibition by Nucleoside Analogs: Cellular- and Kinetic Parameters of Efficacy, Susceptibility and Resistance Selection

**DOI:** 10.1371/journal.pcbi.1002359

**Published:** 2012-01-19

**Authors:** Max von Kleist, Philipp Metzner, Roland Marquet, Christof Schütte

**Affiliations:** 1 Department of Mathematics and Computer Science, Free University Berlin, Berlin, Germany; 2 Institute of Computational Science, University of Lugano, Lugano, Switzerland; 3 Architecture et Réactivité de l'ARN, Université de Strasbourg, CNRS, IBMC, Strasbourg, France; Max-Planck-Institut für Informatik, Germany

## Abstract

Nucleoside analogs (**NA**s) are used to treat numerous viral infections and cancer. They compete with endogenous nucleotides (dNTP/NTP) for incorporation into nascent DNA/RNA and inhibit replication by preventing subsequent primer extension. To date, an integrated mathematical model that could allow the analysis of their mechanism of action, of the various resistance mechanisms, and their effect on viral fitness is still lacking. We present the first mechanistic mathematical model of polymerase inhibition by **NA**s that takes into account the reversibility of polymerase inhibition. Analytical solutions for the model point out the cellular- and kinetic aspects of inhibition. Our model correctly predicts for HIV-1 that resistance against nucleoside analog reverse transcriptase inhibitors (NRTIs) can be conferred by decreasing their incorporation rate, increasing their excision rate, or decreasing their affinity for the polymerase enzyme. For all analyzed NRTIs and their combinations, model-predicted macroscopic parameters (efficacy, fitness and toxicity) were consistent with observations. NRTI efficacy was found to greatly vary between distinct target cells. Surprisingly, target cells with low dNTP/NTP levels may not confer hyper-susceptibility to inhibition, whereas cells with high dNTP/NTP contents are likely to confer natural resistance. Our model also allows quantification of the selective advantage of mutations by integrating their effects on viral fitness and drug susceptibility. For zidovudine triphosphate (AZT-TP), we predict that this selective advantage, as well as the minimal concentration required to select thymidine-associated mutations (TAMs) are highly cell-dependent. The developed model allows studying various resistance mechanisms, inherent fitness effects, selection forces and epistasis based on microscopic kinetic data. It can readily be embedded in extended models of the complete HIV-1 reverse transcription process, or analogous processes in other viruses and help to guide drug development and improve our understanding of the mechanisms of resistance development during treatment.

## Author Summary

Nucleoside analogs (**NA**s) represent an important drug class for the treatment of viral infections and cancer. They inhibit DNA/RNA polymerization after being incorporated into nascent DNA/RNA, which prevents primer extension. Viruses are particularly versatile and frequently develop mutations enabling them to avert the effects of **NA**s. The mechanisms of resistance development are, however, still poorly understood. Through mathematical modeling, we assess the mechanisms by which HIV-1 can develop resistance against nucleoside analog reverse transcriptase inhibitors (NRTI). We quantify the effects of treatment and estimate the fitness of drug resistant mutants. We correctly predict that HIV-1 can develop resistance by decreasing NRTI incorporation rate, increasing its excision rate, or decreasing its affinity for the viral polymerase enzyme. Our model also allows quantification of the cell specific factors affecting NRTI efficacy. Resistance development also changes drug susceptibility distinctly and we show, for the first time, that selection of drug resistance can occur in particular target cells. This finding could provide an explanation of how *clinically* observed resistant viral mutants may arise. It also pin-points important parameters that may impact clinical efficacy of **NA**s used to treat other viruses.

## Introduction

Viral encoded polymerases perform essential enzymatic steps through amplification- or transformation of the viral genome during the viral life cycle [1]. As such, viral encoded polymerases constitute an attractive drug target for the treatment of many viral infections [2]. Nucleoside analogs (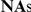) were among the first polymerase inhibitors that showed *clinical* efficacy [3]–[5] and are nowadays broadly used to treat hepatitis B-, herpes simplex- and HIV-1 infection [2], where they constitute the typical backbone components of modern highly active antiretroviral treatment (HAART). Nucleoside analogs are typically formulated as pro-drugs, which require intracellular phosphorylation to form an analog of (deoxy-) nucleoside-triphosphate (NA-TP; mimicking either adenosine, thymidine, guanine, cytosine or uracil), which can be incorporated into nascent viral DNA by the viral polymerase. After incorporation, nucleoside analogs bring the polymerization machinery to a halt, as they lack the chemical group that is necessary to attach the next incoming nucleotide [6]. Incorporated 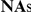 can, however, be selectively excised by some viral polymerases, rescuing the nascent viral DNA and inducing a transient-, rather than permanent mode of inhibition. Inhibition of the crucial step of viral DNA polymerization can lower the probability by which circulating virus can successfully infect host cells [7] and the number of viral progeny produced per unit time, shifting the balance between viral clearance by the immune system and viral replication in favor of the immune system. For the ease of notation, we will subsequently only refer to the active (tri-phosphorylated) nucleoside analog moiety.

Inhibition of DNA polymerization by 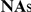 is not restricted to viral polymerase, but can also affect cellular polymerases, leading to unwanted side-effects [8], [9]. The therapeutic window of 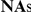 largely depends on molecular kinetic properties of the respective enzymes with regard to a particular inhibitor [10], [11]. 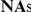 therefore require high specificity for the targeted viral enzyme to allow for a clinical benefit. Viral resistance development can revert this specificity by changing the kinetic properties of the viral enzyme [12], [13]. While a number of enzymatic studies have revealed crucial insights into the mechanisms of polymerase inhibition by 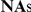 and the kinetic consequences of resistance development, an integrated mathematical insight into these mechanisms has rarely been achieved. In this study, we aim to mathematically formulate a model of polymerase inhibition by 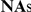, by integrating available enzymatic knowledge. The derived mathematical model should subsequently allow us to assess the impact of distinct cellular- and molecular determinants of 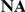 inhibition and to achieve a greater understanding of viral resistance development and epistatic interactions. Results will be exemplified for inhibition of DNA polymerization during reverse transcription (RT) of HIV-1 by nucleoside analog reverse transcriptase inhibitors (NRTIs).

Initial mathematical modelling efforts in the context of RT inhibition by NRTIs of HIV-1 were based on the assumption that incorporation of chain-terminating nucleoside analogs is permanent [14]. The effect of NRTIs was therefore solely explained by their incorporation probability. In subsequent years after the introduction of ziduvudine (AZT; the first NRTI against HIV-1), resistant strains were detected which displayed increased removal kinetics of AZT from terminated primers [15]–[17], rather than discriminating between the natural nucleotide and AZT [18]. This indicated that nucleoside analog removal is very significant and constitutes a major resistance pathway against thymidine analogs (like AZT) and many other NRTIs [13]. The particular mechanism of resistance to AZT indicated that chain termination by nucleoside analogs may not be permanent. Hence, a distinct view on polymerase inhibition by NRTIs is necessary, which departs from the assumption of permanent chain termination. Subsequent modeling work [19] used lumped kinetic expressions and Monte-Carlo simulations instead of deriving analytical expressions, which precludes the identification of key molecular determinants of efficacy and drug resistance. Both previous mathematical modeling efforts were not able to compute the fitness loss associated with mutations in the RT enzyme, an important determinant in clinical settings and for studying epistatic interactions [20]–[23].

In this work, we present a distinct view of viral polymerase inhibition by NRTIs, which departs from the assumption of permanent chain termination. We propose that NRTIs delay the process of DNA polymerization, rather than permanently terminating it, simultaneously keeping in mind that any delay of the process decreases the number of viral progeny and the likelihood of target cell infection by the virus. The developed mathematical formulation allows us to study viral polymerase inhibition by NRTIs as well as fitness effects related to drug resistance development. By integrating fitness effects and drug susceptibility, it is further possible to quantify the selective pressure exerted by NRTIs and to study epistasis. The derived analytical expressions can be used to study the effects of single- and multiple NRTIs on DNA polymerization in the absence and presence of resistance mutations and can be useful for drug design. Chain termination by 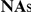 may also be reversible in other viruses [24]–[26], against which 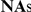 are being developed. Hence, the model may also be applicable to study 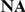 inhibition of these viruses.

## Results

### Mechanism of action of nucleoside analogs on DNA polymerization

**Figure 1:**
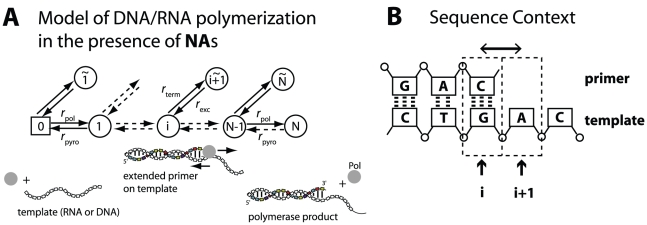
DNA-polymerization in the presence of chain terminating nucleoside analogs. A: The mathematical model defines a Markov jump process: Each state in the model corresponds to the number of incorporated nucleotides: state ‘0’ corresponds to the polymerase enzyme binding to the template, prior to polymerization, states 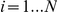 in the model correspond to the condition in which 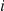 nucleosides have been attached and state 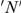 corresponds to full-length product, after which the enzyme dissociates from the template/primer. States 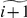 correspond to the condition, in which a DNA-chain consisting of 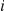 natural nucleosides has been produced, but where the last 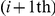 nucleoside in the chain is a chain-terminating 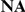. At each state 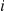, the nascent DNA-chain can either be shortened (pyrophosphorolysis 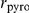), -prolonged with a nucleoside (polymerase reaction 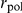) or -terminated by a nucleoside analog (reaction 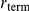). If the chain has been terminated (state 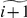), it can get released with rate 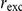 (excision reaction) to produce a chain of length 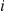. B: Sequence context. The reaction rates 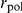, 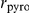, 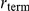 and 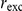 depend on the nucleoside sequence of the template. In the illustration, the next incoming nucleoside could be either a thymidine or a thymidine-analog (corresponding to position 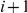 in the template sequence). Therefore, 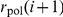 and 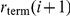 would refer to thymidine- and thymidine-analog incorporation. The pyrophosphorolysis reaction, on the other hand, would refer to cytosine removal (position 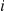 in the primer sequence).

A schematic view of the process of viral DNA polymerization in the presence of 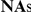 is illustrated in Fig. 1. We interpret the process of DNA polymerization as a Markov jump process with 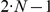 states (Fig. 1A), where each state in the model corresponds to the number of incorporated nucleosides: state ‘0’ corresponds to the initiation of polymerization, states 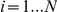 in the model correspond to the condition in which 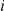 nucleosides have been attached and state 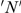 corresponds to the final polymerization product. States 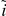 correspond to the condition, in which the DNA-chain consists of 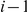 natural nucleosides, but where the last (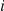
th) molecule in the chain is a chain-terminating nucleoside analog.

At each state 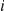, the nascent DNA-chain can either be shortened (pyrophosphorolysis reaction 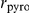), -prolonged with a nucleoside (polymerase reaction 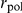) or -terminated by a nucleoside analog (reaction 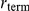). If the chain has been terminated (state 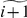), it can get released with rate 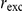 (excision reaction) to produce a chain of length 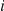. The kinetics of these reactions will be detailed later.

Taking into account the mode of action of chain terminating nucleoside analogs, we conclude that polymerization will be decelerated in the presence of these inhibitors, because the overall time required to go from state ‘0’ (initiation of polymerization) to state 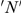 (final polymerization product) in Fig. 1 will be prolonged in their presence by introducing ‘waiting states’ 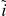. The residual polymerase activity of the wildtype enzyme in the presence of activated (tri-phosphorylated) nucleoside analogs 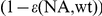 can thus be expressed as:
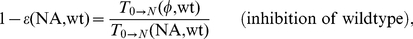
(1)where 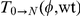 and 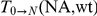 denote the expected time to finalize DNA polymerization in the wildtype 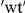 in the absence of drugs 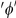 and in the presence of active nucleoside analogs 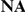 respectively.

Analogously, we can define the effect of chain terminating nucleoside analogs on some viral mutant, 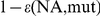 and the fitness loss associated with some mutant in the absence of treatment 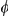, 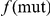:
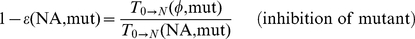
(2)

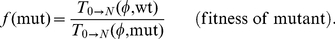
(3)These constituents can be seen as building blocks for describing the fitness landscape of any arbitrary viral mutant 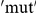 in the absence- and presence of inhibitors, see e.g. [7], [27].

Based on the definitions above, we can also assess the combined effects of selection and drug pressure for any viral strain, i.e. 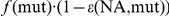. This allows us to assess the selective advantage 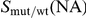 of a mutant viral strain over the wild type in an environment that is pharmacologically modified by 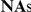.
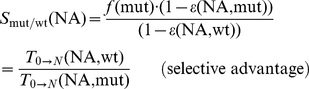
(4)This parameter integrates the (usually opposed) effects of mutations on resistance and viral fitness. If 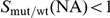, the wild type virus is selected over the mutant strain, whereas 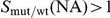 indicates selection of a mutant virus over the wild type. Since 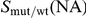 depends on the concentration of 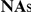, a critical concentration of nucleoside analog 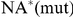 can exist, above which the selection of a particular viral strain over the wild type is favored. 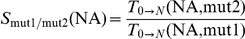 can also be used to assess selection between two arbitrary mutant strains 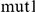 and 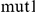 in a pharmacologically modified environment.

Finally, we can assess epistatic interactions for combinations of mutations with regard to viral replication. Briefly, in a two-locus-two-allele model, epistasis is positive if some double mutant m12 replicates better than expected from the single mutants m1 and m2, normalized by the replication of the wild type wt (background). Epistasis is negative if the replication of the double mutant is less than expected from the single mutants. Along the same lines, epistasis has been used to study interactions of mutations in the absence of drugs [22] and for escalating drug concentrations [23]. Using the definitions above, in the presence of 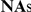, we derive:
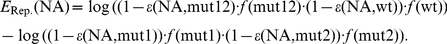
(5)The equation above becomes positive if the first term is greater that the second, i.e. the double mutant replicates better than expected from the single mutants, in agreement with the definition of epistasis [22], [23]. The epistasis term 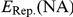 defined above regards both fitness effects and drug resistance. In the absence of drugs, 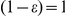, see eqs. (1)–(2) above, we get the fitness epistasis:
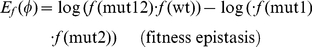
(6)It is also possible to only analyze epistatic effects on resistance:
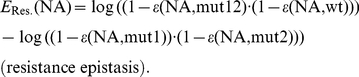
(7)Note, that the defined terms are additive, i.e. 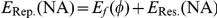.

### Polymerization of Hetero-Polymeric sequences

The process of DNA polymerization (Fig. 1) defines a birth-death process. We are interested in the derivation of an explicit formula for the *mean first passage time* 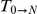 (the average time required to finalize DNA polymerization). Let 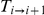 denote the expected time required to extend the DNA-chain by one nucleoside (going from state 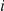 to state 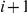, derivation see eq (22)–(28); Methods section)
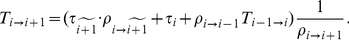
(8)where 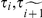 are the waiting times in states 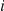 and 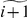 respectively and 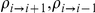 are the probabilities to jump from state 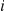 to state 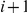 and to state 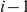 respectively. The parameter 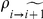 denotes the probability that the chain of length 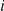 gets terminated by incorporation of a nucleoside analog (state 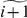). The waiting times 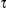 and jump-probabilities 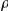 are defined as follows:
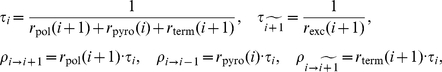
(9)where 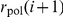 and 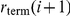 denote the polymerase- and chain terminating reactions (attachment of the next incoming nucleoside or its analog), which depend on the efficacy of incorporation of the respective types of nucleosides (deoxyadenosine, -thymidine, -guanine or -cytosine triphosphate) or their respective analogs at position 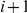 in the primer, see Fig. 1B. The parameter 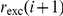 denotes the rate of release (excision reaction) of a primer that has been terminated at position 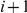 by 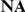. The parameter 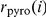 denotes the pyrophosphorolysis reaction, i.e. the rate at which a nucleoside is removed from the end of the primer. Note, that 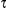 and 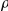 depend on the sequence context because the rates of nucleoside attachment and -removal depend on the types of nucleosides (and -analogs) to be incorporated and -removed respectively (see e.g. Fig. 1B). Eq. (8) allows us to calculate the time to finalize polymerization recursively, using the relation:
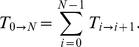
(10)If 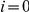 corresponds to the unextended primer, we have 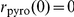 in eq. (9) and therefore eq. (8) simplifies to
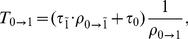
(11)with 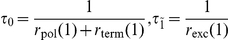 and 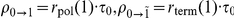, which can be used as a recursion start to compute the polymerization time.

In the case where no chain-terminating inhibitor is applied, we have 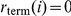 for all 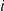 in eq. (9) and therefore eq. (8), and eqs. (10)–(11) simplify accordingly.

Eq. (8)–(10) can subsequently be used to estimate the residual polymerase activity in the presence of 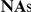 in the wild type and any mutant enzyme, using eq. (1) and eq. (2) respectively, to estimate the fitness of some mutant with regard to polymerization, using eq. (3), or to estimate the selective advantage of a viral strain against a competitor, using eq. (4). This will be exemplified in the next section.

#### Sequence dependent DNA-polymerization in the presence of 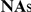


Using eq. (10), it is possible to compute the average polymerization time (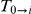) in the absence- and presence of 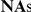 for any arbitrary sequence to be polymerized. In this section, we motivate the use of this approach and show how key phenotypic characteristics can be derived from this simple mathematical model.


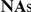
 compete with the natural nucleoside substrates for the same binding site on the polymerase enzyme. We therefore take into account competitive inhibition for the kinetics of nucleoside- and nucleoside analog incorporation.
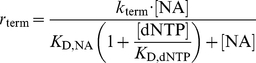
(12)

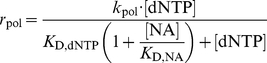
(13)where 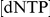 is the concentration of the deoxynucleoside triphosphates (adenosine-, thymidine-, cytidine- and guanosine-) of which the 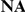 is an analog of. The variable 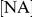 denotes the concentrations of activated (tri-phosphorylated) nucleoside analog that competes with its natural nucleoside counterpart for incorporation into the nascent viral DNA. The parameters 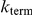 and 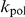 denote the catalytic rate constants for incorporation of the 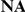 and the dNTP respectively. 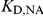 and 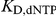 denote the dissociation constants for 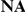 and 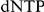 binding to the polymerase respectively. In the absence of inhibitors 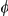, we have 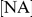 = 0 and therefore eq. (13) and eq. (12) simplify accordingly:
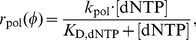
(14)

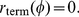
(15)Physiological dNTP concentrations for the most important target cell types of HIV-1 are indicated in Table 1. Parameters for natural nucleoside DNA- and RNA- dependent polymerization by wild type HIV-1 reverse transcriptase (RT) are indicated in Table S1 (supplementary material). In the forthcoming example, we will analyze the effect of a chain-terminating adenosine analog (ddATP, the active metabolite of didanosine, ddI) at a fixed concentration on both single nucleotide incorporation 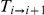 (see eq. (8)) and on cumulative nucleoside polymerization 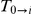 (see eq. (10)) for physiological dNTP concentrations in resting 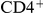 T-cells (Table 1). Furthermore, we will assess how polymerization is impaired by the (clinically relevant) ‘K65R’ mutation in reverse transcriptase in the absence- and presence of ddATP.

**Table 1:** Physiological dNTP levels in different cell types.

	activated 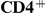 -cells	resting 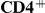 -cells	macrophages	ref.
dATP	5.1	1.7	0.023	[34]
dTTP	7.9	1.5	0.019	[34]
dCTP	5.9	1.9	0.03	[34]
dGTP	4.5	1.7	0.032	[34]
PPi	79	8	7	[35]
ATP	1400	2200	1600	[35]

All values are expressed in 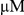.

**Figure 2:**
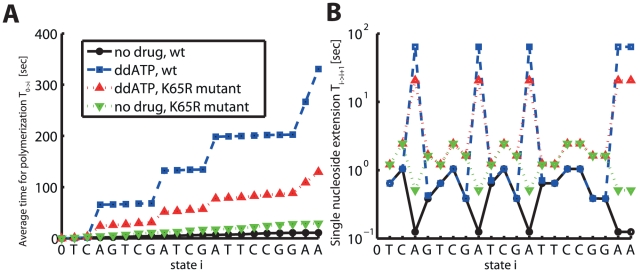
DNA-dependent polymerization of a hetero-polymeric sequence by HIV-1 RT in the presence- and absence of a chain terminating adenosine analog (ddATP). A: Cumulative time for polymerization of a hetero-polymeric sequence in the presence of a chain-terminating nucleoside analog (ddATP). The solid black line (filled dots) indicates the cumulative polymerization time up to sequence position i (the sequence position is indicated at the x-axis) in the absence of inhibitors in the wild type enzyme (calculated using eq. (10)). The dashed blue line (open squares) indicates the time required for polymerization in the presence of 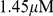 ddATP. The dotted red- and green lines (upward and downward pointing triangles) show the time required for polymerization in the ‘K65R’ mutant RT enzyme in the presence- and absence of 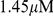 ddATP. Kinetic parameters are presented in Table 1 and Table S1, S2 (supplementary material) for the wild type and the ‘K65R’ mutant. B: Single nucleoside incorporation time 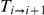 in the absence of ddATP in the wildtype and the ‘K65R’ mutant (solid black and dashed green lines respectively) and in the presence of ddATP in the wild type enzyme (dashed blue line) and in the mutant enzyme (dotted red line), calculated using equation eq. (8).

In Fig. 2 we have computed the average polymerization time for a short sequence (indicated on the x-axis in Fig. 2) and typical parameters for DNA-dependent polymerization for HIV-1 RT, see Table 1 and Table S1 (supplementary material). In this example, we have assumed that 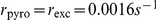 [17] for all dNTP and for ddATP respectively. We examine polymerization in the absence- or the presence of 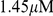 intracellular ddATP. The solid black line denotes the polymerization time in the wild type RT in the absence of ddATP, whereas the blue dashed- and the red dotted lines indicate the polymerization time in the presence of ddATP in the wild type and drug-resistant mutant enzyme (bearing the ‘K65R’ mutation) respectively. The fold changes in the kinetic parameters, induced by the ‘K65R’ mutation, are stated in Table S2 (supplementary material). In the wild type enzyme the predicted incorporation probability 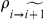 for ddATP over dATP is 9.4% in the presence of 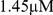 ddATP. For the ‘K65R’ mutant 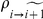 it is 3.2%. In Fig. 2A one can see the cumulative time to form the polymerization product 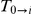. In the presence of ddATP, the cumulative polymerization time is substantially increased (dashed blue line), which is partly compensated in the drug resistant enzyme bearing the ‘K65R’ mutation (dotted red line). In Fig. 2B we show the single nucleoside polymerization time 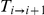. It can be seen, that in the presence of ddATP the single nucleoside polymerization time 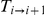 is substantially elevated, in relation to the wild type, whenever the respective natural nucleoside (here adenosine) needs to be incorporated (the solid black line vs. the dashed blue line). In the ‘K65R’ mutant (red dotted line), this is partially compensated for. However, in the mutant, the single nucleoside polymerization time 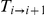 for incorporation of other nucleosides is also increased, which indicates, that the ‘K65R’ mutant might decrease the fitness of the enzyme. We have calculated the fitness of the mutant enzyme, the residual polymerase activity in the wild type enzyme -and the ‘K65R’ mutant and the selective advantage of the ‘K65R’ mutant over the wild type for the presented example, using eqs (1)–(4). The derived values are stated in Table 2. It can be seen that the ‘K65R’ mutant decreases ddATP inhibition of DNA dependent polymerization substantially (the residual polymerization is increased from 3.3% to 22.3%). However, the predicted fitness of the enzyme (in terms of DNA-dependent polymerization) is reduced to 37.9%. The predicted selective advantage of the ‘K65R’ mutant is 2.55, indicating that the ‘K65R’ resistance would be selected over the wild type in the presence of 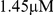 ddATP.

**Table 2:** Efficacy & fitness.

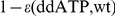	3.31%
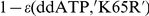	22.3%
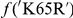	37.9%
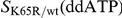	2.55

Residual DNA-dependent polymerase activity 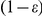 of HIV's RT in resting 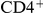 T-cells in the presence of 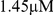 ddATP and fitness 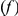 and selective advantage 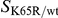 with regard to DNA polymerization for the ‘K65R’ mutant. Calculations are based on formulas (1)–(4).

Note, that in this section, we have exemplified the effects of a particular 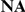 on polymerization, given a specific concentration of the respective 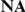 and certain kinetic attributes of the polymerase enzyme (wild type RT vs. ‘K65R’ mutant RT). In the next sections, we will assess the general impact of certain resistance mechanisms, by analyzing a range of kinetic parameters and we will also study the efficacy of 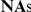 for different concentration ranges.

### Molecular determinants of inhibition

While in a hetero-polymeric sequence context, polymerase inhibition by 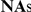 depends on the *particular* succession of the nucleosides, see e.g. Fig. 2B, this is not the case for homo-polymeric sequences, which consist of only one type of nucleoside, e.g. poly-adenosine; ‘Poly-A’. This allows us to derive a general, analytical expression for polymerase inhibition by 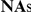, which is valid for *any* homo-polymeric sequence. We will make use of this fact to highlight key determinants of inhibition. For assessing the impact of nucleoside analogs in a *particular* hetero-polymeric sequence context, we advice to use eqs. (8)–(11). In a homo-polymeric sequence, we have 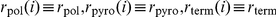 and 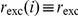 for all 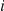. In this particular case, the explicit solution for the *mean first passage time* 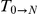 reads (see eq. (31)–(32); Methods section)
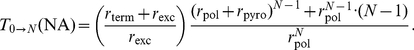
(16)When no inhibitor is present 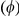, we have 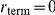 and thus eq. (16) simplifies accordingly:
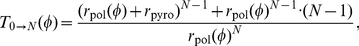
(17)where 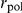 and 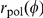 are the polymerization rates in the presence- and absence 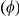 of a competing 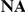, given in eq. (13) and eq. (14). Recalling the effect of 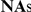 on polymerization, see eq. (1), we can derive the **residual polymerase activity** during 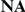 treatment on a **homo-polymeric sequence**, 
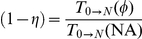
:
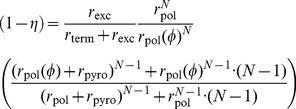
(18)The above expression simplifies further, if the pyrophosphorolysis reaction is very inefficient relative to polymerization, which is the case for most viral polymerase enzymes; e.g. 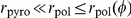.
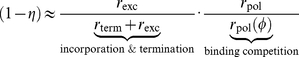
(19)


Eq. (19) highlights the two distinct mechanisms by which inhibition can be conferred, namely a) inhibitor incorporation (and subsequent quasi-termination of the polymerization reaction) and b) competition for binding with natural nucleoside substrates. The efficacy of quasi-termination of the nascent DNA chain depends on the efficacy of inhibitor incorporation 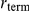 and the duration of the chain termination, determined by 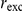. Binding competition is solely determined by the fractional decrease of the natural polymerization reaction (relative to the absence of inhibitor), see eq. (13).

After substituting the enzymatic rate expressions eqs. (12)–(14) into equation (19), we can solve for the fifty percent inhibitory concentration 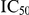 (see eqs. (33)–(35); Methods section), which refers to polymerase inhibition in a homo-polymeric sequence (e.g. ‘Poly-A’) and to the intracellular concentration of activated (triphosphorylated) 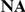.
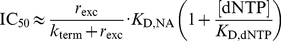
(20)The above equation highlights the processes, which determine the efficacy of a chain-terminating nucleoside analog, namely the kinetic constants 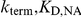 and 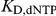, the concentration of natural nucleoside 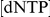 and the excision rate of the inhibitor 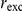.

#### Cell-specific susceptibility to chain-terminating nucleoside analogs

Viruses can infect numerous activated- and resting cells. HIV-1, for example, has been shown to infect activated- and resting 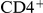 T-cells, macrophages, dendric cells, natural killer cells and microglial cells [28]–[32], and possibly many more. It is important to understand- and take into account heterogeneous- or cell specific drug efficacy, as it may be a primary source of residual viral replication and subsequent resistance development during treatment [33].

**Figure 3:**
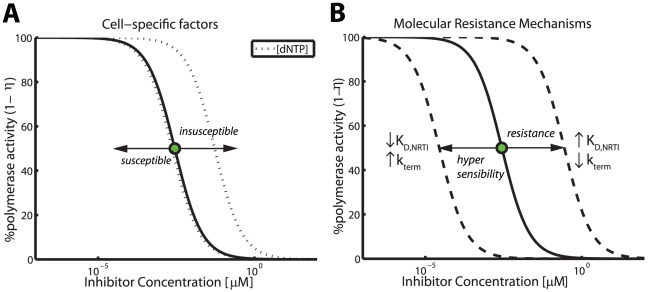
Factors that modify inhibition of DNA polymerization by nucleoside analogs. A: Cell-specific factors: Concentration response curve of ddATP for wild type RT during DNA-dependent polymerization (homo-polymeric sequence) in unstimulated 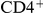 T-cells (solid line) and the impact of a 100-fold variation of the the intracellular nucleoside concentrations (dotted line). The illustration was generated by evaluating eq. (19) and typical parameters for DNA-dependent polymerization during HIV-1 reverse transcription and its inhibition by ddATP (all parameters are indicated in Table 1 and Table S1, supplementary material). The corresponding 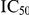 is depicted by a green filled circle. B: Molecular mechanisms of drug resistance and hyper-susceptibility (dashed lines). Impact of (i) selective attrition of inhibitor incorporation 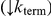 and (ii) selective attrition of inhibitor binding to the primer-template 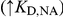 on drug susceptibility. Hypersusceptibility is conferred by the opposite change in the indicated parameters. In order to generate the dashed lines, the respective parameters have been increased/decreased by a factor of 100.

In the context of nucleoside analog efficacy, the major cell-specific factors (apart from pharmacokinetics), are cell type-, or cell stage specific dNTP pools (see Table 1) and possibly cell specific rates of excision 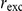. In Fig. 3A, we predicted the impact of cell-specific 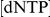 contents on DNA-dependent polymerization during HIV-1 reverse transcription in the presence of ddATP, using typical kinetic parameters (see Table S1, supplementary material).

Under the parameters used, a 100 fold increase in dNTP concentrations would result in a 19 fold increase in the 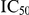 value (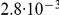 vs. 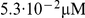), whereas a 100-fold decrease in the dNTP concentrations would only result in a 1.2 fold reduction in the 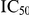 value. This is an important observation, because it indicates that cells that contain high concentrations of dNTP can confer natural resistance against NRTIs, whereas cells with low dNTP content, like macrophages [34], do not necessarily confer hypersusceptibility to NRTIs. This phenomenon can be explained from eq. (20): The 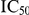 value does not decrease, if 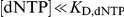.

Resting cells on the other hand might insufficiently phosphorylate NRTIs and subsequently contain lower levels of activated compound. However, these cells do not simultaneously require smaller NRTI concentrations for inhibition (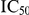 value in Fig. 3A does not decrease with decreasing dNTP levels). Therefore, resting cells could constitute reservoirs for residual replication during antiviral treatment, if NRTI phosphorylation/activation is affected.

Excision of nucleoside reverse transcriptase inhibitors (NRTIs) of HIV-1 from terminated primers has been shown to be mediated by pyrophosphate (PPi) and ATP dependent mechanisms [35]. Whereas ATP concentrations are fairly similar in activated- and resting lymphocytes, as well as macrophages and monocytes [34]–[38] (1 to 5 mM), PPi levels have been shown to vary substantially [35] 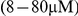, see also Table 1. This indicates that 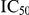 values for polymerase inhibition by 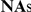 might be cell-specific and may in some cells lead to incomplete suppression. Here, we did not analyze the effect of cell-specific PPi and ATP contents, as the kinetic parameters were not readily available for ddATP. We however discuss their impact on polymerase inhibition by zidovudine (AZT) in a subsequent section.

#### Molecular mechanisms of viral drug resistance against chain-terminating nucleoside analogs

The enzymatic properties of a viral polymerase can be adapted in an evolutionary process to counteract inhibition by 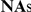. Eq. (20) indicates that the following three distinct molecular mechanisms are likely to induce selective resistance against chain-terminating 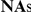, and indeed these three mechanisms of resistance have been described for HIV-1 RT [13].

- selective attrition of inhibitor incorporation (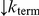)
- selective attrition of inhibitor binding to the primer-template (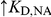)
- enhanced excision of the 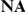 from the terminated primer (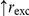, by e.g. increasing the catalytic efficacy of removal or by increasing phosphate-donor, e.g. PPi- or ATP- binding).

The consequences of mutational modification of inhibitor incorporation 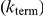 and -binding 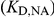 with regard to the predicted efficacy of ddATP are illustrated in Fig. 3B, where we have used typical parameters for DNA-dependent polymerization during HIV-1 reverse transcription (see Table S1, supplementary material). Under the utilized parameters a 100-fold change in the respective parameter 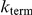 or 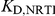 leads to a 100-fold change in the compounds 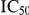 value. We did not analyze the effect of enhanced 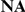 excision in Fig. 3B, as the kinetic parameters were not readily available for ddATP. These effects will be discussed in the context of polymerase inhibition by zidovudine (AZT) in the next section.

### Mechanism of zidovudine (AZT) resistance by thymidine analog mutations (TAMs)

It has been argued [17], that the main mechanism of AZT resistance is due to increased excision of AZT-MP from the terminated primer. In particular, this process has been shown to be both pyrophosphate- (PPi) and ATP- dependent *in vivo* [35]. For the rate of excision 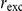 we can therefore write
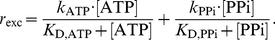
(21)The variables 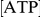 and 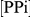 in the above equation refer to the concentration of adenosine triphosphate and pyrophosphate and the parameters 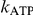 and 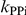 denote the catalytic rate constants for (ATP- and PPi dependent) excision. Parameters 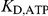 and 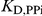 denote the corresponding dissociation constants. The respective concentrations of PPi and ATP in various cell types are shown in Table 1 and kinetic parameters for AZT-MP excision during DNA- and RNA dependent polymerization by HIV-1 RT (wild type and AZT-resistant mutant) are indicated in Table S3 (supplementary material).

#### Residual polymerization in the presence of AZT

**Figure 4:**
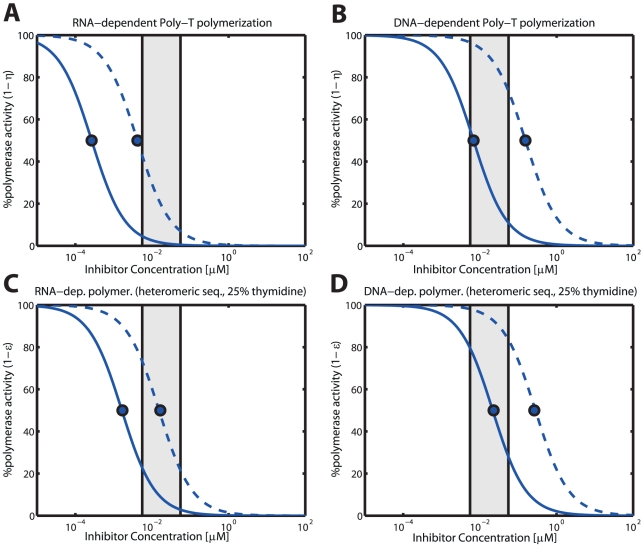
RNA- and DNA-dependent polymerization in the presence of intracellular AZT triphosphate in unstimulated 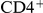 T-cells. The solid blue curves indicate the level of residual polymerization with the wild type enzyme, whereas the dashed lines indicate the residual polymerization with the ‘D67N/K70R/T215Y/K219Q’ mutant. Panels A & B: Residual RNA- and DNA dependent polymerization of a homo-polymeric thymidine sequence (Poly-‘T’). Calculations were obtained by solving eq. (19). Panels C & D: RNA- and DNA polymerization of a hetero-polymeric random sequence of length 500 with 25% respective dNTP content. The illustration was generated using eq. (10). The light grey area indicates the *in vivo* concentrations range of AZT in purified circulating 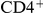 T-cells from [71], converted to units 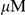 by assuming a cell volume of 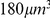 for resting 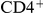 T-cells [72]. All utilized parameters are indicated in Tables 1, S1, S2, S3 (supplementary material).

In Fig. 4, we have illustrated the predicted concentration-response relationship for intracellular AZT triphosphate and RNA- and DNA dependent polymerization of homo-polymeric- (panels A & B) and hetero-polymeric sequences in unstimulated 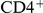 T-cells for the wild type enzyme (solid blue lines) and an AZT-resistant quadruple mutant (‘D67N/K70R/T215Y/K219Q’; dashed lines), respectively. From Fig. 4, several conclusions can be drawn: First, as expected, polymerase inhibition by intracellular AZT is more efficient in homo-polymeric sequences that contain only thymidine versus hetero-polymeric sequences that contain a mixture of all four nucleosides (panel A & B vs. C & D). Second, AZT inhibition of RNA-dependent polymerization is more efficient than inhibition of DNA-dependent polymerization (panels A & C vs. panels B & D). Predicted inhibition of RNA-dependent polymerization of hetero-polymeric sequences is nearly complete for the wild type and under *in vivo* intracellular AZT-TP concentrations (residual activity is 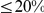, solid blue line and grey area in Fig. 4C). For DNA-dependent polymerization, we predict residual activity under *in vivo* AZT-TP concentrations (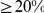, solid blue line and grey shaded area in Fig. 4D). Third, the resistance mutations ‘D67N/K70R/T215Y/K219Q’ (dotted lines) increase the fifty percent inhibitory AZT-TP concentrations. For DNA-dependent polymerization, the 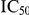 is shifted to concentrations that lie beyond clinically achieved concentrations (see Fig. 4B & Fig. 4D), almost completely diminishing inhibition by AZT (Fig. 4D). RNA-dependent polymerization is still partially inhibited in the ‘D67N/K70R/T215Y/K219Q’ mutant in unstimulated 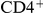 T-cells (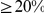 residual polymerization, Fig. 4A & Fig. 4C).

#### Cell type specific susceptibility to AZT and impact of resistance

In Table 3, we have calculated the cell-specific 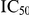 values for RNA- and DNA dependent polymerization of homo-polymeric (Poly-‘T’) sequences. Our results indicate that AZT is much more potent in resting cells (unstimulated 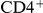 T-cells and macrophages), as suggested by the smaller 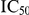 values for the wildtype in Table 3 (second- and fifth column). This cell-specific property is mainly due to lower PPi concentrations in resting cells (see Table 1) and subsequently lesser pyrophosphorolysis of AZT-MP terminated primers in resting cells (see eqs. (20)–(21)) as discussed previously (section *Cell-specific susceptibility to chain-terminating nucleoside analogs*), and is only marginally affected by lower dNTP levels in resting cells, as decreasing dNTP levels may not induce hyper-susceptibility as shown in Fig. 3A. The greatest kinetic change induced by the ‘D67N/K70R/T215Y/K219Q’ affects the catalytic rate of ATP-mediated excision of AZT-MP from the terminated primer 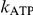 (see Table S3, supplementary material). This change increases the predicted 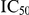 of AZT in unstimulated 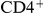 cells and macrophages in a much more pronounced way than in activated 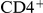 T-cells (fold resistance 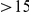 in unstimulated 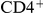 T-cells and macrophages vs. 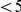 in activated 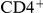 T-cells; fourth and seventh columns in Table 3). In activated T-cells PPi-mediated excision of AZT-MP from the terminated primer is likely the dominant mechanism, as a consequence of the much higher PPi concentrations in these cells (see Table 1). Therefore, increasing 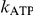 will only have a strong effect once ATP-mediated excision becomes the dominant mechanism of AZT-removal. Therefore, further increase of 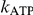 might turn ATP-mediated excision into the main removal pathway and subsequently impact on resistance in a more pronounced way in activated 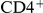 cells as well. Overall, the 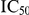 for polymerase inhibition in the ‘D67N/K70R/T215Y/K219Q’ mutant is probably shifted into concentration ranges which are rarely achieved *in vivo*.

**Table 3:** Cell-specific 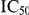 values of AZT-TP for ‘poly-thymidine’ polymerization and susceptibility change by resistance development.

	RNA/DNA			DNA/DNA		
cell type	‘wt’	‘res’*	fold res.	‘wt’	‘res’*	fold res.
act. 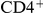	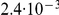	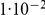	4.5	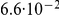	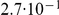	4.1
rest. 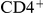	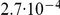	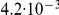	15.7	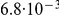	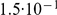	22.6
macr.	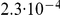	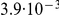	17.2	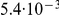	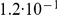	22.5

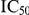
 values, expressed in 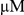, were calculated using eqs. (20)–(21). Cell-specific parameters were taken from Table 1. All kinetic parameters were taken from Table 1 and Tables S1, S2, S3 (supplementary material).

*‘res’ = D67N/K70R/T215Y/K219Q mutant.

#### Molecular mechanism of AZT-resistance by ATP-mediated excision

**Figure 5:**
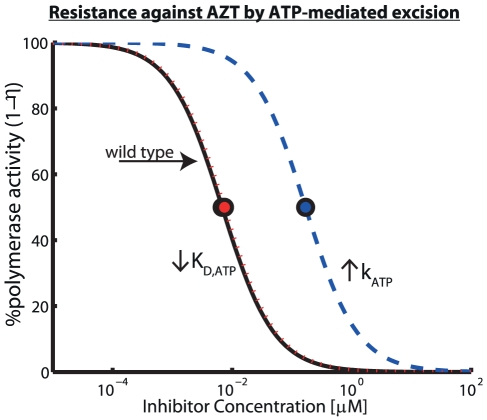
Molecular mechanisms of HIV-1 resistance development against AZT by ATP-mediated excision. Potential mechanisms for resistance development against AZT through increasing its excision rate 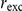 via an ATP-mediated mechanism (see eq. (21)). We calculated residual DNA-dependent polymerization of a Poly-T sequence in unstimulated 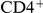 T-cells using eq. (19) with parameters from Tables 1, S1 and S3 (supplementary material). The solid black line shows residual DNA polymerization 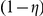 in the wild type virus, whereas the dotted red line and the dashed blue line refer to residual polymerization if 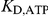 and 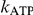 were decreased- and increased 100-fold respectively.

Excision of AZT-MP from the terminated primer is the major mechanism by which AZT resistance is thought to be mediated [17]. In particular, ATP-mediated excision has been discussed as the major *in vivo* mechanism of AZT resistance [15], [16]. However, at the molecular level, it is unclear, if the mechanism by which enhanced excision is achieved is due to an increased removal rate (parameter 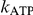 in eq. (21)) or increased binding affinity of ATP to the primer-template (affected parameter: 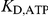 in eq. (21)). In particular, in a recent paper [39], it was argued, based on crystal structures of resistant RT, that the main mechanism of AZT-resistance could be conferred by increasing ATP's binding affinity to the resistant RT enzyme. In Fig. 5, we analyze the impact of the two potential AZT-resistance mechanisms (increased removal rate 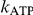 vs. decreased 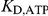). Our predictions show that increasing the affinity for ATP binding 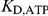 (dashed red line) does not lead to resistance development under the parameters used, because ATP binding to the wild type enzyme is already saturated (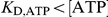) at physiological conditions and further decrease of 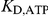 enhances the saturation effect. However, increasing the removal rate 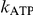 (dashed blue line) desensitizes reverse transcriptase-mediated polymerization to AZT inhibition since 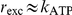, in cells with low PPi contents and under saturation conditions (see Table 1 and eq. (21)).

### Selection of resistance

Selection of drug resistance depends on the competitive advantage of some resistant mutant over its competitors (either the wild type or some competing viral mutant) in a particular environment. In order to quantify whether drug resistant mutants become selected in an environment that is modified by 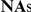, we have previously defined the selective advantage 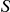 in eq. (4) (and paragraph below).

#### Selection of thymidine associated mutations (TAMs) by AZT in different cell-types

**Figure 6:**
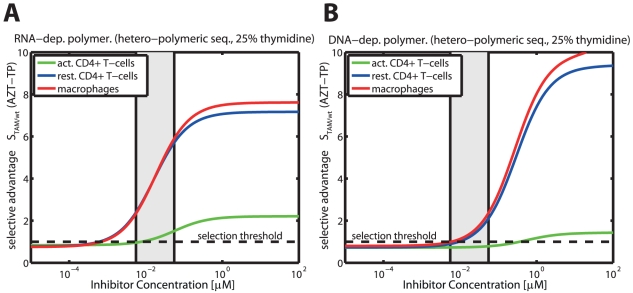
Selective advantage of the ‘D67N/K70R/T215Y/K219Q’ mutant against the wild type during RNA- and DNA-dependent polymerization in the presence of AZT-TP. The solid lines (green = activated 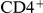 cells, blue = unstimulated 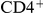 cells, red = macrophages) indicate the selection parameter 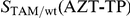, defined in eq. (4), for different levels of intracellular ATZ-TP during RNA- and DNA dependent polymerization (Panels A & B) of a random sequence of length 500 with 25% respective dNTP content. The light grey area indicates the *in vivo* concentrations range of AZT in purified circulating 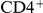 T-cells from [71], converted to units 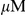 by assuming a cell volume of 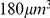 for resting 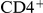 T-cells [72]. The dashed horizontal line indicates the threshold for resistance selection, i.e. 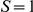. All utilized parameters are indicated in Table 1 and Tables S1, S2, S3 (supplementary material).

In Fig. 6A and Fig. 6B, the selective advantage of TAMs over the wild type 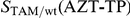 is shown for RNA-dependent polymerization (panel A) and DNA-dependent polymerization (panel B) respectively in distinct cell-types relevant to HIV-1 infection (solid green-, blue and red lines indicate 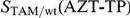 for activated 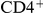 T-cells, resting 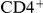 T-cells and macrophages, respectively). The respective threshold concentrations 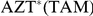 above which resistance becomes selected, 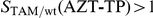, are 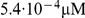 (resting 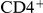 cells) 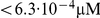 (macrophages) 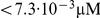 (activated 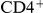 cells) for RNA-dependent polymerization. For DNA-dependent polymerization, the corresponding thresholds are 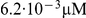 (macrophages) 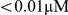 (resting 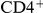 cells) 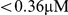 (activated 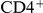 cells).

Two major findings can be derived from Fig. 6: Firstly, it can be seen that in the case of RNA-dependent polymerization, the ‘D67N/K70R/T215Y/K219Q’ mutation becomes selected (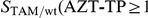; dashed horizontal black line) at lower intracellular AZT-TP concentrations (below clinically achieved concentrations in resting 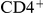 T-cells and macrophages; light grey area) compared to DNA-dependent polymerization. During DNA-dependent polymerization, ‘D67N/K70R/T215Y/K219Q’ is only selected at clinically relevant levels of AZT-TP (resting 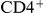 T-cells and macrophages) or far above (activated 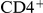 T-cells). We have shown previously in Fig. 4C & D that inhibition of RNA-dependent polymerization by AZT-TP is much more efficient compared with inhibition of DNA-dependent polymerization (see also Table 3), explaining the higher selective pressure exerted at lower AZT-TP concentrations during RNA-dependent polymerization. Therefore, we would expect that resistance is favored at lower concentrations during RNA-dependent polymerization, when compared to DNA-dependent polymerization.

Secondly, and quite surprisingly, Fig. 6A & B indicate that resistance to AZT may not become selected over the wildtype in activated 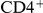 cells as it only confers a very small selective advantage in these cell types during RNA-dependent polymerization and at clinically relevant concentrations of AZT-TP (solid green line and grey area in Fig. 6A). For DNA-dependent polymerization the selection parameter indicates a disadvantage (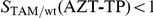) of the ‘D67N/K70R/T215Y/K219Q’ mutant at clinically relevant AZT-TP concentrations. In resting 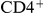 T-cells and macrophages on the other hand, resistance selection is favored at clinically relevant AZT-TP concentrations (DNA-dependent polymerization) and below (RNA-dependent polymerization). These results indicate, that selection of the ‘D67N/K70R/T215Y/K219Q’ mutation by AZT is cell-specific and may preferably occur within resting 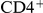 T-cells and macrophages, whereas resistance selection in activated 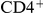 T-cells is less likely. This finding, however, warrants further investigation of the intermediate strains in the TAM resistance pathway, once kinetic data becomes available.

#### Subsequent selection of Q151M-complex mutations by TDF

**Figure 7:**
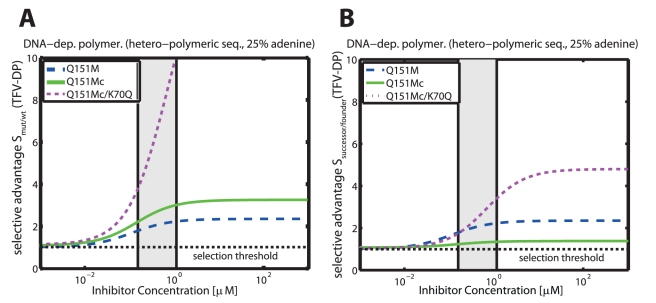
Selective advantage 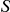 of intermediate viral mutants of the Q151M-complex during DNA-dependent polymerization in the presence of TFV-DP. Dashed blue-, solid green- and dotted magenta line indicate the selective advantage of the Q151M, the multi-drug resistant Q151M-complex (Q151Mc: A62V/V75I/F77L/F116Y/Q151M) and the Q151Mc+K70Q mutation during DNA-dependent polymerization of a random sequence of length 500 with 25% respective dNTP content in unstimulated 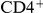 cells. The light grey area indicates the *in vivo* concentrations range of TFV-DP from [56], [71], [73], converted to units 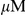 by assuming a cell volume of 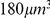 for resting 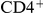 T-cells [72]. The dashed horizontal line indicates the threshold for resistance selection, i.e. 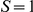. Panel A: Selective advantage of the respective mutants with regard to wild type 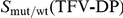. B: Selective advantage of a succeeding mutants with regard to progenitor in Q151M complex formation 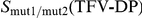. All utilized parameters are indicated in Table 1 and Tables S1, S2 (supplementary material).

The selective advantages of intermediate viral strains of the Q151M-complex (multi-drug) resistance pathway (Q151M, A62V/V75I/F77L/F116Y/Q151M (Q151Mc) and Q151Mc/K70Q ) with respect to increasing tenofovir diphosphate (TFV-DP) concentrations are shown in Fig. 7 for DNA-dependent polymerization in resting 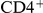 T-cells. Panel A shows the selective advantage of the respective mutant in relation to the wild type, i.e. 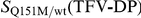 (dashed blue line), 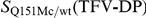 (solid green line) and 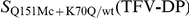 (dotted magenta line). At *in vivo* concentrations ranges of TFV-DP (light grey area) the selective pressure towards the Q151M and the Q151Mc strains is relatively weak 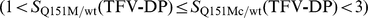, whereas it is strong for the Q151Mc/K70Q mutant 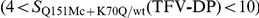. It can be seen that the selective advantage is of the order 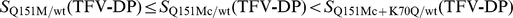, indicating a distinctly graded ‘selection landscape’ from the wild type towards the Q151Mc/K70Q mutant. A graded landscape would imply that the presence of TFV-DP favors subsequent resistance mutations in the resistance pathway. We therefore further analyzed the form of the ‘selection landscape’ in panel B, where we have plotted the selective advantage of the respective mutants in relation to their progenitors in the resistance pathway, i.e. 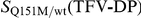, 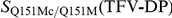, 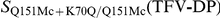. It can be seen that the Q151M single mutation has a weak selective advantage over the wild type (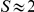 dashed blue line). The Q151M-complex (Q151Mc) has an even weaker selective advantage over the Q151M single mutation in the presence TFV-DP (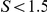, solid green line). However, the subsequent mutation, 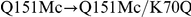 has a strong selective advantage in the presence of TFV-DP 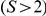. The selection landscape therefore exhibits a slight increase 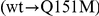, followed by a plateau 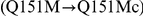, followed by a steep increase 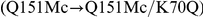. Our analysis indicates that TDF treatment slightly favors Q151M over the wild type, it, however, does not favor the Q151M-complex 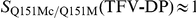. Once the Q151M-complex has arisen (due to co-administered drugs), TDF could select for the K70Q mutation.

### Epistasis

**Figure 8:**
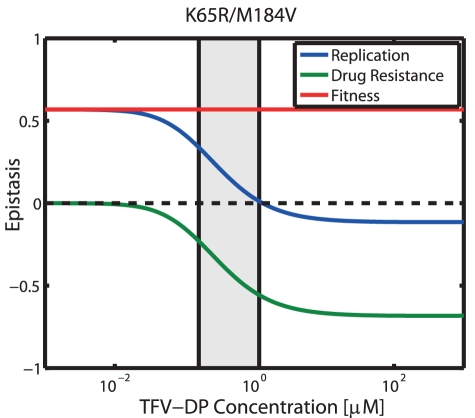
Epistatic interactions for DNA-dependent polymerization in the presence of TFV-DP. Solid blue-, green- and red line indicate epistasis with regard to replication 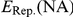, resistance 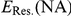 and fitness 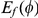 as defined in eqs. (5)–(7) for the double mutant ‘K65R/M184V’. The black dashed horizontal line indicates the value, where no epistatic interactions occur. The light grey area indicates the *in vivo* concentrations range of TFV-DP from [56], [71], [73], converted to units 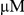 by assuming a cell volume of 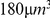 for unstimulated 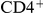 T-cells [72]. All utilized parameters are indicated in Table 1 and Tables S1, S2 (supplementary material).

Epistasis has been used to describe the phenomenon where the phenotype of one mutation is modified by one or several other mutations [22], [23]. In a two-locus-two allele model, epistasis is said to be positive when the combined effects of a double mutant result in greater replication than expected if the effects on replication coming from the two single mutations were independent. Conversely, epistasis is said to be negative, when the combined effects of a double mutant result in lesser than expected replication. Resistance mutations against NRTIs of HIV-1 are located within the same gene (the *Pol* gene). It is therefore likely, that the combination of mutations produce a phenotype that has unexpected/novel properties. The intention of this analysis is to elucidate how epistasis depends on the environment in which the virus replicates (and which is altered by 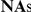), analogously to [23]. In Fig. 8, we assessed epistasis with regard to replication (solid blue line), fitness (solid red line) and resistance (solid green line), based on eqs. (5)–(7) for the K65R/M184V mutant and varying TFV-DP concentrations for DNA-dependent polymerization in resting 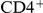 T-cells.

It can be seen that epistasis in the absence of drugs 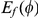 (fitness epistasis) is positive (solid red line). This result is based on the fact that the predicted fitness of the double mutant 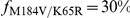 is larger than expected if the fitness effects coming from the respective single mutants 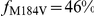 and 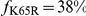 were independent. Resistance epistasis 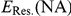 (green line) on the other hand is negative at clinically relevant TFV-DP concentrations (light grey area). Whereas the M184V mutation is slightly hypersusceptible (predicted fold resistance relative to the wild type: 0.76 see also [40]), the K65R mutation confers 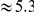-fold resistance in relation to the wild type, mainly by decreasing TFV-DP's incorporation rate 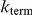, see Table S2 (supplementary information). We predicted that the double mutant ‘M184V/K65R’ is 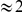-fold resistant in relation to the wildtype. Resistance epistasis 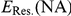 thus reduces replication of the double mutant in the presence of TFV-DP and is negative. The combined effects of fitness and drug resistance are indicated by the blue line in Fig. 8. Our predictions indicate that epistasis is positive at clinically relevant TFV-DP concentrations (light grey area), because the (positive) fitness epistasis overweighs the negative resistance epistasis in the clinically relevant range of TFV-DP concentrations. At higher TFV-DP concentrations, however, the negative resistance epistasis overweighs.

### Residual DNA-dependent polymerization of mutant reverse transcriptase (RT) of HIV-1 in the presence of distinct nucleoside reverse transcriptase inhibitors (NRTIs)

Viral fitness is an important determinant for the pre-treatment abundance of drug resistant mutants and their persistence in circulating virus after withdrawal of drugs. Moreover, it has also important implications for the therapeutic strategy and on disease progression [20], [21]. For these reasons, we assessed viral fitness of the distinct mutants in the absence of drugs. We estimated viral fitness on the basis of the relative decrease in polymerization time, see eq. (3), for a hetero-polymeric sequence context and based on DNA-dependent polymerization during reverse transcription. The results are presented in Table 4 (bottom row). The fitness of the viral mutants was of the order 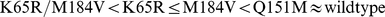 and is in general agreement with published data on viral fitness [21], [41]. Notably, the K65R and M184V mutants conferred substantial fitness losses, which explains the low prevalence of K65R even in treatment experienced patients [21], and M184V reversion to wild type when 3TC, ABC or FTC are eliminated from second or third-line anti-retroviral regimens [42].

**Table 4:** Estimated *in vivo* % residual DNA-dependent polymerization 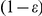 for distinct mutants and drug combinations.

	wt	Q151M	M184V	K65R	M184V/K65R
TFV-DP	4.16–24.11	9.20–42.55	3.32–20.05	19.11–63.34	8.72–41.14
AZT-TP	29.47–80.69	-	-	-	-
d4T-TP	2.08–25.95	-	7.44–56.97	-	-
FTC-TP	2.07–21.45	1.24–14.00	-	21.28–77.76	47.37–92.09
3TC-TP	1.54–4.95	0.86–2.81	51.22–77.71	12.29–31.77	86.19–95.40
CBV-TP	7.63–14.18	82.27–90.27	45.49–62.53	-	-
FTC-TP	1.39–12.69	1.11–11.75	-	11.02–53.12	7.80–39.22
+TFV-DP					
d4T-TP	0.91–4.40	-	7.01–49.20	-	
+3TC-TP					
CBV-TP	1.33–3.89	0.87–2.84	32.20–53.53	-	-
+3TC-TP					
CBV-TP	1.27–3.81	-	-	-	-
+3TC-TP					
+AZT-TP					
fitness	100	100	46	38	30

*In vivo* concentration ranges were 3TC-TP = 12.2–40.5; FTC-TP = 1.5–19.4; TFV-DP = 0.16–1.17; CBV-TP = 0.44–0.88; d4T-TP = 0.034–0.56; and AZT-TP = 0.0056–0.056 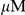 respectively [56], [71], [73]–[75], assuming an average cell volume of 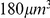 for resting 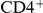 T-cells [72].

Estimated residual DNA-dependent polymerization for mutant and wild type RT under *in vivo* concentration ranges of triphosphorylated NRTIs in resting 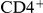 T-cells and on a hetero-polymeric sequence context (using eqs. (1)–(2)) are presented in Table 4. Utilized kinetic parameters for nucleoside incorporation are provided in Table S2 (supplementary material). We predicted that most inhibitors decreased DNA-dependent polymerization to values of 2–25% in the wildtype enzyme. However, 3TC displayed superior efficacy (only 1.5–5% residual polymerization) and AZT only poorly inhibited DNA-dependent polymerization. However, as discussed in section *Residual polymerization in the presence of AZT*, AZT is likely to exert its main effect through inhibition of RNA-dependent polymerization. The Q151M mutation decreased the efficacy of carbovir triphosphate (CBV-TP) markedly (8 fold) and had only marginal impact on tenofovir diphosphate (TFV-DP), whereas lamivudine triphosphate (3TC-TP) and emtricitabine triphosphate (FTC-TP) were unaffected (see also [40], [43]). Combination treatment with 3TC-TP+CBV-TP could, however, restore inhibition of polymerization and combination treatment FTC-TP+TFV-DP was very efficient, however not markedly different from FTC-TP alone. The M184V mutation decreased susceptibility to 3TC-TP (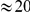 fold) and CBV-TP (8 fold), having marginal impact on stavudine triphosphate (d4T-TP) and no effect on TFV-DP, which is consistent with phenotypic measurements [40], [43]. Susceptibility to the combination of d4T-TP+3TC-TP was comparable to d4T-TP alone. The efficacy of 3TC-TP+CBV-TP was strongly reduced. We predicted that the K65R mutation reduced the impact of 3TC-TP, FTC-TP and TFV-DP (7-, 4 and 3-fold respectively) and also reduced the susceptibility to the combination FTC-TP+TFV-DP (5-fold), consistent with phenotypic measurements [40], [43]. The double mutation K65R/M184V conferred complete resistance to 3TC-TP and near complete resistance to FTC-TP and partly restored susceptibility to TFV-DP or TFV-DP+FTC-TP, compared to K65R alone, in agreement with phenotypic measurements [40], [43].

### Inhibition of human mitochondrial polymerase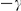 by various NRTIs

Despite their antiviral activity, NRTIs have been reported to cause severe mitochondrial toxicity [9], [44], limiting their therapeutic use. A dominant hypothesis for the manifestation of mitochondrial toxicity by NRTIs is that NRTIs inhibit polymerase-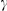 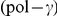 function, which is necessary to duplicate the mitochondrial genome, thereby leading to mtDNA depletion and subsequent mitochondrial abnormalities. The anticipated mechanism of 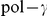 inhibition is highly similar to inhibition of polymerization during reverse transcription: tri-phosphorylated NRTIs compete with endogenous dNTPs for incorporation into the nascent mtDNA, and, once incorporated, lead to quasi-chain termination [9]. Polymerase-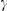 can perform two crucial catalytic functions, namely DNA polymerization and exonuclease activity; the later enabling the removal of incorporated NRTIs. The mechanism of action of NRTIs on 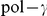 leads us to believe that our mathematical model of polymerase inhibition by 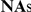 can be useful in predicting NRTI-induced inhibition of 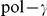.

Utilizing pre-steady state kinetic data for the incorporation of dNTPs and various NRTIs (see Table S4, supplementary material), we estimated the residual 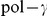 function in a hetero-polymeric sequence context and under concentration ranges of NRTI-TPs typically observed *in vivo*. The results are stated in Table 5. For simulation purposes we utilized eqs. (1) and assumed dNTP levels typically observed in unstimulated 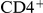 cells (see Table 1). Under the parameters used, we found that mtDNA polymerization is substantially inhibited in the presence of d4T-TP and moderately inhibited by 3TC-TP for *in vivo* -triphosphate concentration ranges. Similarly, combinations 3TC-TP+D4T-TP reduced 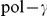 activity substantially and 3TC-TP+CBV-TP or 3TC-TP+AZT-TP+CBV-TP reduced 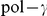 activity moderately. We found the following order of inhibition of polymerase-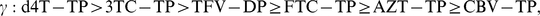 which agrees with experimental findings [9]. The mitochondrial toxicity of AZT is likely not due to 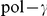 inhibition. Instead, it has been explained in terms of various other mechanisms, which are exemplified in the *Discussion* section.

**Table 5:** Estimated *in vivo* % residual human mitochondrial polymerase-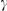 activity in resting 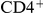 cells.

	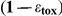	ther. Index*
TFV-DP	63.54–92.72%	5.5
AZT-TP	98.74–99.87%	†
d4T-TP	0.15–2.40%	0.1
FTC-TP	94.05–99.51%	8.2
3TC-TP	25.69–53.43%	12.2
CBV-TP	98.78–99.38%	9.1
FTC-TP/TFV-DP	61.96–92.55%	11
3TC-TP/d4T-TP	0.16–2.48%	0.5
CBV-TP/3TC-TP	25.18–52.70	14.9
CBV-TP/3TC-TP/AZT-TP	26.22–54.13%	†

*In vivo* concentration ranges were 3TC-TP = 12.2–40.5; FTC-TP = 1.5–19.4; TFV-DP = 0.16–1.17; CBV-TP = 0.44–0.88; d4T-TP = 0.034–0.56; and AZT-TP = 0.0056–0.056 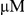, respectively [56], [71], [73]–[75], assuming an average cell volume of 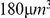 for resting 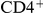 T-cells [72].

*calculated as the ratio of average effect on polymerase-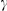 and wildtype reverse transcriptase of HIV-1: 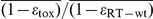.

**†:** mitochondrial toxicity of AZT has been attributed to mechanisms other than 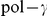 inhibition (see *Discussion* section).

We subsequently defined a therapeutic index as the ratio of the mean inhibition of 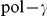 and wild type RT respectively. The therapeutic index indicated the following order for the inhibitors and their combinations: . Note, that AZT has been excluded from this assessment, because its mitochondrial toxicity has been contributed to mechanisms other than 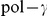 inhibition (see *Discussion* section).

## Discussion

We presented a novel mechanistic mathematical model of HIV-1 polymerase inhibition by 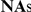 that, for the first time, focussed on the transient aspect of this inhibition. This is an important characteristic, as HIV-1 can exploit the transient nature of inhibition by reducing the residence time of the apparent chain terminator (the incorporated 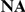) in the nascent viral DNA to achieve drug resistance (summarized in [13]). 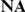 removal from quasi-terminated RNA/DNA chains has also been described for hepatitis B & C viruses [24]–[26]. Hence, the developed model may also be applicable to study polymerase inhibition by 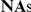 in these viruses. In contrast to previous mathematical approaches [14], [19], we therefore describe the effects of nucleoside analogs on DNA-polymerization in terms of an increase in the average polymerization time, which is analogous to a reduction of the overall polymerization rate, i.e. 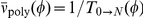. This mathematical approach not only allows to study various resistance mechanisms, but also allows for the first time to estimate the inherent fitness of drug resistant mutants, resulting from microscopic changes in the polymerization rate constants (e.g. 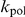, 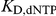) of the mutant viral enzyme (see eqs. (1)–(3)). The derived model can readily be used to assess the probability to successfully finish polymerization. In supplementary Text S1 we have given an example for HIV-1 reverse transcription. It is also explained therein how the model can be integrated in larger (systems biology) models of the viral life cycle in order to study the effects of 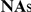.

The developed model can be parameterized in terms of physiological parameters (such as dNTP concentrations) and microscopic kinetic rates (e.g. 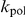, 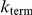, 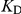), typically derived from cell-free *in vitro* assays. These parameters can usually be precisely determined with standard errors 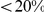. We demonstrated the applicability of the model for various distinct polymerization processes, in particular for polymerase inhibition during HIV-1 RT and mitochondrial 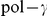 by NRTIs, respectively. Adaptation to distinct polymerization processes was achieved by utilizing the kinetic constants for the respective processes, while the model remained unchanged. Notably, model-predicted macroscopic predictions (viral fitness, drug efficacy and toxicity) were consistent with various experimental macroscopic findings and thus underline the usefulness of the proposed model.

Based on the developed model of polymerization and its inhibition by 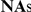, we derived two sets of mathematical solutions: Eqs. (1)–(11) can be used to compute the average effect of 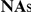 and combinations of 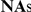 on polymerization of arbitrary (hetero-polymeric) DNA sequences. Analogously, these equations can be used to determine the deceleration of polymerization resulting from resistance mutations in the absence of any 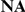, as an indicator of their inherent fitness cost. On the other hand, eqs. (16)–(20) represent analytical solutions for polymerase inhibition by 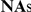 in a simplified homo-polymeric sequence context. The resulting equations (19)–(20) immediately highlight key determinants of 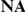 inhibition and resistance development in this context. These equations can also be used to determine the model's sensitivity for different combinations of kinetic- and physiological parameters, see Fig. 3 and Fig. 5. Based on eqs. (19)–(20), we found that factors impacting on 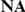 inhibition can generally be divided into two categories: (i) kinetic- and (ii) cellular factors.

Eq. (20) revealed that the rate of 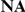 incorporation 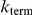, its binding affinity 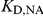 and the catalytic rate of 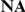 removal 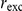 are key molecular kinetic determinants for the efficacy of 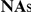. All indicated molecular kinetic determinants (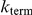, 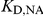 and 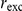) depend on the viral polymerase enzyme and are thus prone to resistance development. The impact of alterations in these parameters is illustrated in Fig. 3B for ddATP and in Fig. 4 & 5 for AZT-TP.

Various reports indicate cell-specific differences in 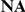 efficacy against HIV-1 [45]–[47]. Differences in efficacy were often brought in association with intracellular NA-TP∶dNTP ratios [48], [49]. Utilizing the derived model, we elucidated the impact of cellular factors on HIV-1 RT polymerase inhibition by NRTIs. Quite surprisingly, we found that cells that contain low dNTP content do not necessarily confer hypersusceptibility to NRTIs if 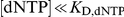 (see Fig 3A). For AZT, we predicted that alteration of PPi and ATP levels can have a strong impact on its efficacy (see Table 3). In summary, we demonstrated that the concurrence of multiple kinetic- and physiological factors, rather than a single parameter, can determine the susceptibility of an infected cell towards 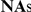, see eq. (20)–(21). In addition to cells that contain an unfavorable NA-TP∶dNTP ratio [48], [49], cells that contain high levels of PPi or ATP and low levels of 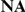 (regardless of their dNTP content) could be resistant to NRTI treatment and residual viral replication despite treatment could persist in these cells as well. This finding can have important consequences for HIV-1 treatment with NRTIs, as HIV-1 exhibits a broad cell tropism [28]–[32]: While some evidence for low-level ongoing replication in the context of apparently suppressive antiviral therapy has been reported [50], the cellular source remains to be determined [51]. Whereas it has been shown previously [33], that heterogeneous viral inhibition facilitates drug resistance development, we show evidence for cell-specific (thus heterogeneous) inhibition by NRTIs. Thus, a possible mechanism for the emergence of drug resistance against could be explained on the basis of the mechanism of action of these compounds. However, further evidence is required to confirm this hypothesis.

We analyzed the specific mechanisms of AZT resistance through TAMs. It is well known, that TAMs induce resistance through increasing the excision of incorporated 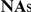 from nascent viral DNA. However, the precise mechanism that increases excision is controversial. A recent crystal structure of resistant RT [39], showed that the orientation of ATP is altered in the mutant enzyme. Based on this structure [39], the authors argued that ATP, which serves as an excision substrate for incorporated AZT, would bind with higher affinity to the quasi-terminated nascent viral DNA, accelerating the removal of incorporated AZT. To the contrary, our kinetic model indicated that increasing the affinity for ATP binding 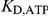 does not lead to resistance development (see Fig. 5), because ATP binding to the wild type enzyme is already saturated (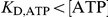) at physiological conditions, and further decrease of 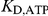 enhances the saturation effect. Increasing the removal rate 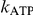 desensitizes reverse transcriptase-mediated polymerization to AZT inhibition since 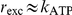, in cells with low PPi contents and under saturation conditions (see Table 1 and eq. (21)). We therefore propose that the main kinetic resistance effect of the altered orientation of ATP in mutant RT is mediated by an increased removal rate 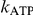, in agreement with a pre-steady state kinetic analysis [17], although binding could be affected. In particular, the crystal structure showed that the resistance mutations affect the positioning of ATP in the RT catalytic site [39], which must translate into an effect on 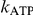.

We quantified the inhibitory effects of AZT during RNA- and DNA dependent polymerization and we analyzed how TAMs (‘D67N/K70R/T215Y/K219Q’) induce susceptibility changes. We found that AZT inhibition during HIV-1 reverse transcription is more efficient during RNA-dependent polymerization than during DNA-dependent polymerization, see Fig. 4. Moreover, inhibition, as well as susceptibility changes induced by TAMs were found to be cell-specific (see Table 3).

While the emergence of a particular viral strains depends on a) the probability that the mutant is generated (related to residual replication and genetic distance), it also critically depends on the likelihood that the generated mutant becomes selected subsequently. However, if inhibition- and selection forces are different in distinct target cells (see Table 3 and Fig. 6), then the processes of mutant strain generation and subsequent selection might also we divided among target cells. We therefore further looked at the selective advantage 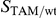 of the ‘D67N/K70R/T215Y/K219Q’ mutant in distinct cell types. Specifically, we predicted that the selective advantage of the ‘D67N/K70R/T215Y/K219Q’ mutation in the presence of AZT at clinically relevant concentrations is quite distinct in activated 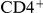 cells, resting 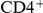 cells and macrophages (see Fig. 6). We found that the ‘D67N/K70R/T215Y/K219Q’ mutation is less likely selected over the wild type in activated 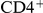 cells, whereas this mutation is preferred in resting 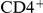 cells and macrophages (see Fig. 6) at clinically relevant concentrations. While these results indicate, for the first time, that selection forces against 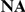 treatment can be quite distinct for diverse target cells, a detailed analysis of the various intermediate mutants in the TAM resistance pathway is required, in particular a construction of the ‘selection landscape’ for particular mutants in the resistance pathway and for different cell types infected with HIV-1 in the presence of combinations of drugs to fully understand resistance dynamics *in vivo*. The developed model can be used to facilitate such an analysis: In Fig. 7, we started to reconstruction the ‘selection landscape’ for intermediate mutants of the Q151M-complex during TDF treatment in unstimulated 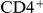 cells. We found for this cell type, that TDF alone is unlikely to select the Q151M-complex over the Q151M single mutation. Once the Q151M-complex has arisen, however, TDF would select for the additional K70Q mutation. An extended analysis of the resistance pathways in the case where particularly large genetic barriers are involved may in the future help to understand and influence the dynamics of resistance emergence for e.g. TAMs and the Q151M complex.

Epistasis has been suggested as a method to study evolutionary dynamics of virus populations [52]. It describes the phenomenon where the replicative fitness of one mutation is modified by one or several other mutations [22], [23]. Epistasis is said to be positive when the combined effects of two-or-more mutations result in greater replication than expected if the effects coming from the two single mutations were independent. Since resistance mutations against NRTIs of HIV-1 are located within the same enzyme (RT), several mutations could modify the enzyme in unexpected ways, i.e. result in epistatic interactions with regard to fitness and resistance. We have shown in Fig. 8 that our model can be used to analyze different aspects of epistasis (fitness, resistance and replication). In the presented example, we detected positive fitness epistasis 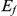 of the ‘M184V/K65R’ double mutant and negative resistance epistasis 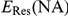 with increasing TFV-DP concentrations in comparison with the single mutations. The combined effects of fitness- and resistance were positive at relevant concentration ranges of TFV-DP. The major conclusion from this analysis is that the combination of mutations can alter the RT enzyme in unexpected ways. The phenotypic attributes of a multiple mutated strain may not be intuitively related to the attributes of the single mutants. It is thus required to view each multiple mutated strain as an independent entity with regard to resistance and fitness. For deriving information about intermediate viral mutants in a resistance pathway (e.g. the Q151M-complex, or TAMs), it is therefore necessary to measure the attributes of each intermediate strain independently. Related experimental work [23] indicated that replication ranking, rather than epistasis predicts dynamics of resistance emergence, in line with our analysis in section *“Selection of Resistance”*.

Based on the developed model, we predicted that the ‘D67N/K70R/T215Y/K219Q’ mutation induces a 4.1 to 22.6 fold increase in the 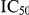 value for poly-thymidine polymerization, depending on the cell type and the template (RNA or DNA). In cellular assays, the ‘D67N/K70R/T215Y/K219Q’ mutant can induce a 120–150 fold increase in the fifty percent inhibitory (extracellular) concentration when measured in 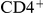 HeLa-cells [46] and a 8000 fold increase in MT-4 human T-lymphoid cells [47], respectively, while at the same time resistance at the enzymatic level was observed to be far more moderate [47]. This indicates that a direct quantitative comparison of susceptibility changes observed in different cell-based assays and changes computed at the enzymatic level, e.g. on the basis of DNA-dependent polymerization in resting 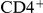 cells (see Table 3) might not be possible. Here, we summarize a few mechanisms, which could contribute to this difference: (i) Firstly, the cell types utilized in distinct cell-based assays differ, which can results in distinct susceptibility changes to NRTIs. We discussed- and illustrated the impact of these cell-specific differences in *Cell type specific susceptibility to AZT and impact of resistance* and in Table 3 for AZT. For AZT, these cell-specific differences were attributed to different contents of PPi and dTTP. (ii) Secondly, two different outputs are measured by the two methods: In contrast to RT activity, phenotypic assays measure the production of viral proteins, which denotes a step in the viral life cycle following polymerization and reverse transcription of the viral genome. (iii) Thirdly, and most importantly, the 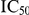 values based on enzymatic activity (as computed in this work) refer to intracellular concentrations of AZT-triphosphate, while the fold change derived by cell-based assays refers to the concentrations of extracellular pro-drug (AZT) added to the medium surrounding the cells. This has important consequences: AZT phosphorylation is known to be non-linear and might be saturated at the bottlenecking step of thymidilate kinase 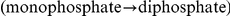 [53], [54]. We have shown previously that the *in vivo* maximally achievable AZT-TP concentration is close to the clinically achieved AZT-TP concentration in peripheral blood mononuclear cells (PBMCs), when 300 mg AZT is given twice daily, see [53]. In order to disproportionately increase the 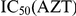 value several hundred-fold, as observed with some mutants e.g. ‘M41L/D67N/K70R/T210W/Y215F’, at the enzymatic level all that is required is a minor fold change in the 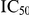 (for AZT-TP), that shifts the fifty percent inhibitory concentration of intracellular AZT-TP beyond the maximally achievable levels. Thus, by adding more extracellular AZT, sufficient concentrations of AZT-TP may never be reached. In the case of saturating intracellular AZT monophosphate (AZT-MP) concentrations, the cell-specific levels of thymidilate kinase enzyme will ultimately determine the maximally achievable AZT-TP concentration, which are therefore also cell-specific [55].

In Table 4 we analyzed, based on the developed model, how different mutations can specifically alter the efficacy of distinct NRTIs and their combinations on DNA-directed polymerization and at physiological concentrations. Estimated susceptibility changes resulting from distinct mutations were *qualitatively* in good agreement with results from cell culture assays (see [40]), although, as mentioned earlier, it should be noted that a direct *quantitative* comparison of our estimations with results from cell-culture assays may not be possible. While estimating the effect of combinations of 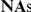 on DNA polymerization is straightforward using eq. (1)–(11), we did not assess clinically relevant pharmacokinetic interactions between different 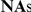. Pharmacokinetic interactions between NRTIs of HIV-1 have mainly been attributed to interactions during the cellular activation cascade [56]. For our estimations in Table 4 we therefore assessed only drug combinations that use distinct enzymes in their phosphorylation cascade and which therefore bear lesser potential for pharmacokinetic interaction than drugs which utilize the same intracellular phosphorylation pathway.

Inhibition of mitochondrial polymerase-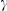 by NRTIs has been proposed as a central process for their clinical toxicity [9]. We therefore studied inhibition of polymerase-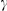 by distinct NRTIs at physiologically relevant triphosphate concentrations. The ranking of polymerase-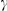 inhibition by the analyzed NRTIs was in good agreement with published results [9], indicating a strong inhibition of 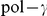 by d4T and moderate inhibition by 3TC at physiological intracellular triphosphate concentrations. However, it should also be noted, that mitochondria in different tissues might contain different levels of dNTP and NRTI-TPs and might therefore be differentially prone to 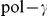 inhibition, potentially contributing to site-specific toxicities of some NRTIs [9]. Mitochondrial toxicity of AZT has been explained by other mechanisms than 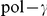 inhibition. In particular, AZT might deplete dNTP pools in the mitochondria, rather than quasi-terminate nascent mtDNA by its incorporation [57], [58].

Although we demonstrated the use of the developed model on nucleoside reverse transcriptase inhibitors of HIV-1 throughout the article, we did not construct a mathematical model of the complete reverse transcription process, but rather focussed on the sub-process of polymerization, which is primarily targeted by NRTIs and other 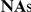. The aim was to point out general principles of inhibition and resistance development, rather than establishing customized models for the respective targeted viral processes. Therefore, the presented model can be used to also assess effects on distinct polymerase enzymes, or as demonstrated in Table 5 to assess off-target effects of 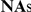. Furthermore, the model can readily be used to assess inhibition of polymerization by NcRTIs, a novel class of pre-marketed nucleoside inhibitors which compete with natural dNTPs for binding to the polymerase enzyme, without becoming incorporated [59]–[61].

In the future, the developed model could be extended for the “dead-end complex”-mechanism observed during inhibition of HIV-1 RT [13], if respective kinetic parameters become available. Extension of the model is straightforward, as it only requires the introduction of an additional state in the mathematical model (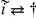 in Fig. 1) and the subsequent derivation of the corresponding equations, analogously to the derivations in this article.

Recent *in vitro* experiments with single-molecules of HIV-1 RT indicated that additional complexities might occur during the reverse transcription process, such as enzyme-template dissociation and association and reversal of orientation to perform distinct tasks, such as RNAse H cleavage of the viral RNA template [62], [63]. While these results warrant further investigation, it has been shown that *in vivo* an excess of RT (50–200 enzymes/virion) in comparison to RNA template may be present [64], such that different enzymes could perform different tasks (polymerization/RNAse H) at the same time *in vivo*. The cooperativity of multiple RT enzymes can also explain the distinct shape of the dose-response curve observed in primary human cells with inhibitors that directly target the enzyme, such as non-nucleoside reverse transcriptase inhibitors (NNRTIs), in contrast to inhibitors that target the RNA/DNA template (NRTIs) [65], [66]. The development of models of reverse transcription that also incorporate the effects of non-nucleoside reverse transcriptase inhibitors (NNRTIs) [67], [68] warrants further mechanistic understanding of the complex overall process of reverse transcription and will be left for future research. The developed model can however be readily be used to model the effects of NAs and will be further extended to model e.g. the complete reverse transcription process of HIV-1 genomic RNA, or analogous processes in other viruses (see also supplementary Text S1).

## Methods

### Derivation of a recursive solution for the polymerization times on arbitrary hetero-polymeric sequences

In this section we will derive the analytical solution for the polymerization time given in eq. (10), which is based on ideas given in [69]. Recall that the proposed model is a Markov jump process and that the polymerization time 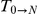 is given by the *mean first passage time* (MFPT) to go from state ‘0’ (initiation of polymerization) to the state 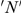 (final polymerization product).

Starting point for the derivation are the MFPT-equations (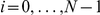) [70],
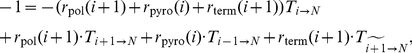
(22)

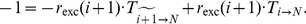
(23)Eq. (23) yields
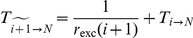
such that eq. (22) simplifies to
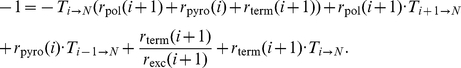
Further algebraic rearrangements yield
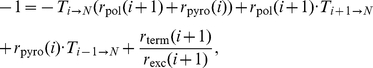
and finally
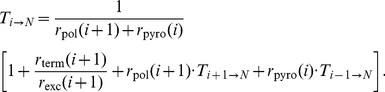
(24)We define the general relation
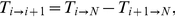
(25)which allows us to express 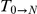 as a telescope sum 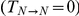, i.e.,
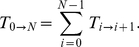
(26)From the general relation (25), we can derive 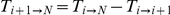 and 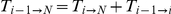, which were substituted into equation (24). Rearrangement produces the recursion
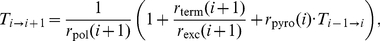
(27)which equals
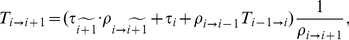
(28)with parameter definitions given in eq. (9) of the main text.

Equation (27) is satisfied by
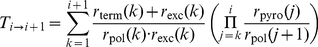
(29)such that the initial condition holds, i.e.,
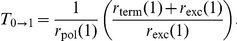
Finally, inserting (29) into (26) results in the analytical expression for 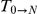,
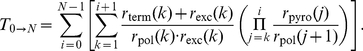
(30)


### Derivation of an analytic solution for polymerization times of homo-polymeric sequences

In case where the sequence to be polymerized is homo-polymeric, e.g. ‘Poly-A’, all rates are uniform, i.e., 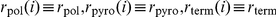 and 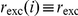 for any 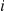. Then by exploiting twice the identity
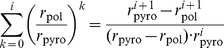
(31)the polymerization time from eq. (30) simplifies to
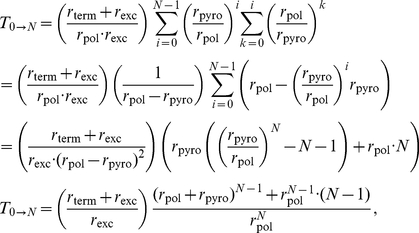
(32)which is displayed in eq. (16) of the main article.

### Determination of the fifty percent inhibitory concentration 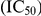


Starting point for calculating the fifty percent inhibitory concentration (for polymerization of uniform sequences) is equation (19). We set
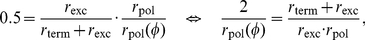
(33)substitute eqs. (12)–(14) and solve for the 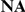 concentration (that yields 50% inhibition, the 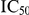 value). After rearranging, we get the quadratic formula
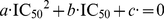
(34)with
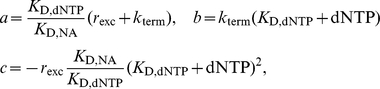
which yields
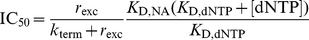
(35)


## Supporting Information

Table S1 **Pre-steady state kinetic constants for nucleoside incorporation by wild type HIV-1 reverse transcriptase.** Indicated parameters are average values from the respective literature sources.
(PDF)

Table S2
**Fold change of kinetic parameters for DNA-dependent polymerization in various HIV-1 reverse transcriptase mutants, relative to wildtype RT.** 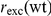 was set to the value of 0.0016 [1/s] in resting 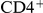 T-cells for thymidine- and adenosine analogs respectively, see Table S3 (supplementary material) and eq. (18) (main article) and to the value of 0.00053 [1/s] for guanine- and cytosine analogs, see [76]. 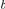 excision of TFV-TP from terminated templates was assumed to be 100%, 50%, 100% and 40% of the wild type excision rate for the M184V, the K65R, the Q151M and the K65R/M184V mutant, see [77]. 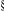 CBV-TP excision in the Q151M mutant was set to 5300% of wild type excision, see [76]. D4T-TP excision in the M184V mutant was set to 83% of the wild type excision, assuming a similar effect of M184V on D4T-TP and AZT-TP [77]. If no other information was available, excisions of nucleoside analogs in the mutant enzymes were assumed to be equal to the wild type excision rate. 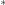 Q151Mc denotes the ‘A62V/V75I/F77L/F116Y/Q151M’ mutant. 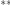 4-TAM denotes the ‘D67N/K70R/T215Y/K219Q’ mutant. 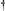 set to the value of 1, because of insufficient information. 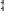 set equal to the rate in Q151Mc.(PDF)

Table S3
**Pre-steady state kinetic constants for AZT excision by HIV-1 reverse transcriptase wildtype and ‘D67N/K70R/T215Y/K219Q’ mutant.** 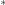 Parameter could not be accurately determined in the respective study [17].(PDF)

Table S4
**Pre-steady state kinetic constants for nucleoside incorporation by human mitochondrial polymerase-** 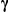 **.** 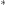 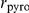 was set to value zero because of insufficient information.(PDF)

Text S1 **The supplementary text contains the modelling required to compute the probability to successfully complete reverse transcription (RT) in HIV-1, based on the parameters presented in the main manuscript.**
(PDF)
