# Supplementary material for: HIV-1 Polymerase Inhibition by Nucleoside Analogs: Cellular- and Kinetic Parameters of Efficacy, Susceptibility and Resistance Selection
Source: PLoS Comput Biol. 2012 Jan 19;8(1):e1002359. doi: 10.1371/journal.pcbi.1002359 (PMC3261923; doi:10.1371/journal.pcbi.1002359)
Supplement: Table S1 — Pre-steady state kinetic constants for nucleoside incorporation by wild type HIV-1 reverse transcriptase. Indicated parameters are average values from the respective literature sources. (PDF) [file pcbi.1002359.s001.pdf]

**Table S1. Pre-steady state kinetic constants for nucleoside incorporation by wild type HIV-1 reverse transcriptase**

| | RNA-dependent | | | DNA-dependent | | |
|---|---|---|---|---|---|---|
| | $K_D$ $\mu$M | $k_{pol}$ $[s^{-1}]$ | ref. | $K_D$ $\mu$M | $k_{pol}$ $[s^{-1}]$ | ref. |
| dATP | 16 | 74 | [1] | 7.8 | 44.8 | [1–3] |
| dTTP | 25.5 | 87 | [4,5] | 15.3 | 15.6 | [2,3,5,6] |
| dCTP | 20 | 22.7 | [3,7–9] | 18.25 | 10.2 | [3,7,8,10] |
| dGTP | 11.5 | 34.5 | [11,12] | 10 | 20 | [11,12] |
| ddATP | - | - | - | 43.9 | 30.75 | [1,2] |
| 3TC-TP | - | - | - | 20.17 | 0.038 | [7,10,13] |
| TFV-DP | - | - | - | 40.5 | 28 | [1,13] |
| CBV-TP | - | - | - | 21 | 1 | [14] |
| FTC-TP | - | - | - | 19 | 0.056 | [10,15] |
| AZT-TP | 22.8 | 54.3 | [3–5] | 7.15 | 1.9 | [3,5] |
| d4T-TP | - | - | - | 34.5 | 13.4 | [6,16] |

Indicated parameters are average values from the respective literature sources.

# References


1. Suo Z, Johnson KA (1998) Selective inhibition of HIV-1 reverse transcriptase by an antiviral inhibitor, (r)-9-(2-phosphonylmethoxypropyl)adenine. J Biol Chem 273: 27250–27258.

2. Selmi B, Boretto J, Sarfati SR, Guerreiro C, Canard B (2001) Mechanism-based suppression of dideoxynucleotide resistance by K65R human immunodeficiency virus reverse transcriptase using an alpha-boranophosphate nucleoside analogue. J Biol Chem 276: 48466–48472.

3. Krebs R, Immendrfer U, Thrall SH, Whrl BM, Goody RS (1997) Single-step kinetics of HIV-1 reverse transcriptase mutants responsible for virus resistance to nucleoside inhibitors zidovudine and 3-TC. Biochemistry 36: 10292–10300.

4. Rigourd M, Lanchy JM, Grice SFL, Ehresmann B, Ehresmann C, et al. (2000) Inhibition of the initiation of HIV-1 reverse transcription by 3'-azido-3'-deoxythymidine. comparison with elongation. J Biol Chem 275: 26944–26951.

5. Kerr SG, Anderson KS (1997) Pre-steady-state kinetic characterization of wild type and 3'-azido-3'-deoxythymidine (AZT) resistant human immunodeficiency virus type 1 reverse transcriptase: implication of RNA directed DNA polymerization in the mechanism of AZT resistance. Biochemistry 36: 14064–14070.

6. Yang G, Wang J, Cheng Y, Dutschman GE, Tanaka H, et al. (2008) Mechanism of inhibition of human immunodeficiency virus type 1 reverse transcriptase by a stavudine analogue, 4'-ethynyl stavudine triphosphate. Antimicrob Agents Chemother 52: 2035–2042.

7. Feng JY, Anderson KS (1999) Mechanistic studies comparing the incorporation of (+) and (-) isomers of 3TCTP by HIV-1 reverse transcriptase. Biochemistry 38: 55–63.

8. Vaccaro JA, Singh HA, Anderson KS (1999) Initiation of minus-strand DNA synthesis by human immunodeficiency virus type 1 reverse transcriptase. Biochemistry 38: 15978–15985.

9. Wilson JE, Aulabaugh A, Caligan B, McPherson S, Wakefield JK, et al. (1996) Human immunodeficiency virus type-1 reverse transcriptase. contribution of met-184 to binding of nucleoside 5'-triphosphate. J Biol Chem 271: 13656–13662.

10. Feng JY, Myrick FT, Margot NA, Mulamba GB, Rimsky L, et al. (2006) Virologic and enzymatic studies revealing the mechanism of K65R- and Q151M-associated HIV-1 drug resistance towards emtricitabine and lamivudine. Nucleosides Nucleotides Nucleic Acids 25: 89–107.

11. Jeffrey JL, Feng JY, Qi CCR, Anderson KS, Furman PA (2003) Dioxolane guanosine 5'-triphosphate, an alternative substrate inhibitor of wild-type and mutant HIV-1 reverse transcriptase. Steady state and pre-steady state kinetic analyses. J Biol Chem 278: 18971–18979.

12. Ray AS, Murakami E, Basavapathruni A, Vaccaro JA, Ulrich D, et al. (2003) Probing the molecular mechanisms of AZT drug resistance mediated by HIV-1 reverse transcriptase using a transient kinetic analysis. Biochemistry 42: 8831–8841.

13. Deval J, White KL, Miller MD, Parkin NT, Courcambeck J, et al. (2004) Mechanistic basis for reduced viral and enzymatic fitness of HIV-1 reverse transcriptase containing both K65R and M184V mutations. J Biol Chem 279: 509–516.

14. Ray AS, Basavapathruni A, Anderson KS (2002) Mechanistic studies to understand the progressive development of resistance in human immunodeficiency virus type 1 reverse transcriptase to abacavir. J Biol Chem 277: 40479–40490.

15. Feng JY, Murakami E, Zorca SM, Johnson AA, Johnson KA, et al. (2004) Relationship between antiviral activity and host toxicity: comparison of the incorporation efficiencies of 2',3'-dideoxy-5-fluoro-3'-thiacytidine-triphosphate analogs by human immunodeficiency virus type 1 reverse transcriptase and human mitochondrial DNA polymerase. Antimicrob Agents Chemother 48: 1300–1306.

16. Selmi B, Boretto J, Navarro JM, Sire J, Longhi S, et al. (2001) The valine-to-threonine 75 substitution in human immunodeficiency virus type 1 reverse transcriptase and its relation with stavudine resistance. J Biol Chem 276: 13965–13974.
