# Supplementary material for: HIV-1 Polymerase Inhibition by Nucleoside Analogs: Cellular- and Kinetic Parameters of Efficacy, Susceptibility and Resistance Selection
Source: PLoS Comput Biol. 2012 Jan 19;8(1):e1002359. doi: 10.1371/journal.pcbi.1002359 (PMC3261923; doi:10.1371/journal.pcbi.1002359)
Supplement: Table S2 — Fold change of kinetic parameters for DNA-dependent polymerization in various HIV-1 reverse transcriptase mutants, relative to wildtype RT. was set to the value of 0.0016 [1/s] in resting T-cells for thymidine- and adenosine analogs respectively, see Table S3 (supplementary material) and eq. (18) (main article) and to the value of 0.00053 [1/s] for guanine- and cytosine analogs, see [76]. excision of TFV-TP from terminated templates was assumed to be 100%, 50%, 100% and 40% of the wild type excision rate for the M184V, the K65R, the Q151M and the K65R/M184V mutant, see [77]. CBV-TP excision in the Q151M mutant was set to 5300% of wild type excision, see [76]. D4T-TP excision in the M184V mutant was set to 83% of the wild type excision, assuming a similar effect of M184V on D4T-TP and AZT-TP [77]. If no other information was available, excisions of nucleoside analogs in the mutant enzymes were assumed to be equal to the wild type excision rate. Q151Mc denotes the ‘A62V/V75I/F77L/F116Y/Q151M’ mutant. 4-TAM denotes the ‘D67N/K70R/T215Y/K219Q’ mutant. set to the value of 1, because of insufficient information. set equal to the rate in Q151Mc. (PDF) [file pcbi.1002359.s002.pdf]

Table S2. Fold change of kinetic parameters for DNA-dependent polymerization in various HIV-1 reverse transcriptase mutants, relative to wildtype RT

	M184V		K65R		K65R/M184V		Q151M		
	K_D	k_{pol}	K_D	k_{pol}	K_D	k_{pol}	K_D	k_{pol}	ref.
dATP	2.4	0.66	0.93	0.23	4.9	0.4	2	1	[1–4]
dTTP	3.5	1.08	0.57	0.33	1.76	0.77	0.82	1.13	[1, 2, 4, 5]
dCTP	2.66	1.3	1.5	0.62	2.4	0.78	1.5	1.2	[1–4, 6]
dGTP	1.75	1.5	1.65	0.37	4.7	1.1	0.52	1.1	[1, 7–10]
	K_D	k_{term}	K_D	k_{term}	K_D	k_{term}	K_D	k_{term}	ref.
3TC-TP	35	0.55	0.77	0.081	17.9	0.035	0.23	0.32	[1, 6]
TFV-DP$^\flat$	1.3	0.98	0.78	0.046	2.87	0.14	1.6	0.34	[1, 3]
CBV-TP§	2.43	0.45	-	-	-	-	0.07	0.11	[8]
FTC-TP	-	-	1.59	0.128	1	0.016	0.32	0.42	[6]
ddATP	-	-	1.4	0.11	-	-	-	-	[1, 2]
AZT-TP	-	-	-	-	-	-	-	-	-
D4T-TP‡	12.6	1.86	-	-	-	-	-	-	[5]

r_{exc}(wt) was set to the value of 0.0016 [1/s] in resting CD4$^+$ T-cells for thymidine- and adenosine analogs respectively, see Table S3 (supplementary material) and eq. (18) (main article) and to the value of 0.00053 [1/s] for guanine- and cytosine analogs, see [8]. $^\flat$ excision of TFV-TP from terminated templates was assumed to be 100%, 50%, 100% and 40% of the wild type excision rate for the M184V, the K65R, the Q151M and the K65R/M184V mutant, see [11]. § CBV-TP excision in the Q151M mutant was set to 5300% of wild type excision, see [8]. D4T-TP excision in the M184V mutant was set to 83% of the wild type excision, assuming a similar effect of M184V on D4T-TP and AZT-TP [11]. If no other information was available, excisions of nucleoside analogs in the mutant enzymes were assumed to be equal to the wild type excision rate.

Table S2. (continued)

	Q151Mc*		Q151Mc/K70Q		4-TAM**		4-TAM(RNA)		
	K_D	k_{pol}	K_D	k_{pol}	K_D	k_{pol}	K_D	k_{pol}	ref.
dATP	3.7	2.41	1.92	2.3	$1^{\dagger}$	$1^{\dagger}$	$1^{\dagger}$	$1^{\dagger}$	[4, 12]
dTTP	0.57	0.58	$0.57^{\ddagger}$	$0.58^{\ddagger}$	0.53	0.24	1.6	0.72	[4, 13]
dCTP	0.82	1.1	$0.82^{\ddagger}$	$1.1^{\ddagger}$	$1^{\dagger}$	$1^{\dagger}$	$1^{\dagger}$	$1^{\dagger}$	[4]
dGTP	4.1	3.8	$4.1^{\ddagger}$	$3.8^{\ddagger}$	0.79	1.15	2.83	1.8	[7, 10]
	K_D	k_{term}	K_D	k_{term}	K_D	k_{term}	K_D	k_{term}	ref.
3TC-TP	-	-	-	-	-	-	-	-	-
TFV-DP	2.3	0.46	4.7	0.36	-	-	-	-	[12]
CBV-TP	-	-	-	-	-	-	-	-	-
FTC-TP	-	-	-	-	-	-	-	-	-
ddATP	-	-	-	-	-	-	-	-	-
AZT-TP	-	-	-	-	3.5	2.57	2.46	0.67	[13]
D4T-TP	-	-	-	-	-	-	-	-	-

* Q151Mc denotes the 'A62V/V75I/F77L/F116Y/Q151M' mutant. ** 4-TAM denotes the 'D67N/K70R/T215Y/K219Q' mutant. † set to the value of 1, because of insufficient information. ‡ set equal to the rate in Q151Mc.

References

1. Deval J, White KL, Miller MD, Parkin NT, Courcambeck J, et al. (2004) Mechanistic basis for reduced viral and enzymatic fitness of HIV-1 reverse transcriptase containing both K65R and M184V mutations. J Biol Chem 279: 509–516.

2. Selmi B, Boretto J, Sarfati SR, Guerreiro C, Canard B (2001) Mechanism-based suppression of dideoxynucleotide resistance by K65R human immunodeficiency virus reverse transcriptase using an alpha-boranophosphate nucleoside analogue. J Biol Chem 276: 48466–48472.

3. Frangeul A, Bussetta C, Deval J, Barral K, Alvarez K, et al. (2008) Gln151 of HIV-1 reverse transcriptase acts as a steric gate towards clinically relevant acyclic phosphonate nucleotide analogues. Antivir Ther 13: 115–124.

4. Deval J, Selmi B, Boretto J, Egloff MP, Guerreiro C, et al. (2002) The molecular mechanism of multidrug resistance by the q151m human immunodeficiency virus type 1 reverse transcriptase and its suppression using alpha-boranophosphate nucleotide analogues. J Biol Chem 277: 42097–42104.

5. Yang G, Wang J, Cheng Y, Dutschman GE, Tanaka H, et al. (2008) Mechanism of inhibition of human immunodeficiency virus type 1 reverse transcriptase by a stavudine analogue, 4'-ethynyl stavudine triphosphate. Antimicrob Agents Chemother 52: 2035–2042.

6. Feng JY, Myrick FT, Margot NA, Mulamba GB, Rimsky L, et al. (2006) Virologic and enzymatic studies revealing the mechanism of K65R- and Q151M-associated HIV-1 drug resistance towards emtricitabine and lamivudine. Nucleosides Nucleotides Nucleic Acids 25: 89–107.

7. Jeffrey JL, Feng JY, Qi CCR, Anderson KS, Furman PA (2003) Dioxolane guanosine 5'-triphosphate, an alternative substrate inhibitor of wild-type and mutant HIV-1 reverse transcriptase. Steady state and pre-steady state kinetic analyses. J Biol Chem 278: 18971–18979.

8. Ray AS, Basavapathruni A, Anderson KS (2002) Mechanistic studies to understand the progressive development of resistance in human immunodeficiency virus type 1 reverse transcriptase to abacavir. J Biol Chem 277: 40479–40490.

9. Ray AS, Yang Z, Shi J, Hobbs A, Schinazi RF, et al. (2002) Insights into the molecular mechanism of inhibition and drug resistance for HIV-1 RT with carbovir triphosphate. Biochemistry 41: 5150–5162.

10. Feng JY, Myrick F, Selmi B, Deval J, Canard B, et al. (2005) Effects of hiv q151m-associated multi-drug resistance mutations on the activities of (-)-beta-d-1',3'-dioxolan guanine. Antiviral Res 66: 153–158.

11. Ly JK, Margot NA, MacArthur HL, Hung M, Miller MD, et al. (2007) The balance between NRTI discrimination and excision drives the susceptibility of HIV-1 RT mutants K65R, M184V and K65R+M184V. Antivir Chem Chemother 18: 307–316.

12. Hachiya A, Kodama EN, Schuckmann MM, Kirby KA, Michailidis E, et al. (2011) K70q adds high-level tenofovir resistance to "q151m complex" hiv reverse transcriptase through the enhanced discrimination mechanism. PLoS One 6: e16242.

13. Kerr SG, Anderson KS (1997) Pre-steady-state kinetic characterization of wild type and 3'-azido-3'-deoxythymidine (AZT) resistant human immunodeficiency virus type 1 reverse transcriptase: implication of RNA directed DNA polymerization in the mechanism of AZT resistance. Biochemistry 36: 14064–14070.
