# Supplementary material for: HIV-1 Polymerase Inhibition by Nucleoside Analogs: Cellular- and Kinetic Parameters of Efficacy, Susceptibility and Resistance Selection
Source: PLoS Comput Biol. 2012 Jan 19;8(1):e1002359. doi: 10.1371/journal.pcbi.1002359 (PMC3261923; doi:10.1371/journal.pcbi.1002359)
Supplement: Table S3 — Pre-steady state kinetic constants for AZT excision by HIV-1 reverse transcriptase wildtype and ‘D67N/K70R/T215Y/K219Q’ mutant. Parameter could not be accurately determined in the respective study [17]. (PDF) [file pcbi.1002359.s003.pdf]

Table S3. Pre-steady state kinetic constants for AZT excision by HIV-1 reverse transcriptase wildtype and 'D67N/K70R/T215Y/K219Q' mutant

	wild type			'D67N/K70R/T215Y/K219Q'			
	DNA	RNA	ref		DNA	RNA	ref
$K_{D,ATP}$ μM	870	100*	[1]	fold change	0.37	1*	[1]
k_{ATP} $[\times 10^{-3} s^{-1}]$	0.56	0.17	[1]	fold change	50	12.9	[1]
$K_{D,PPi}$ μM	970	1800	[1]	fold change	4.3	0.12	[1]
k_{PPi} $[s^{-1}]$	0.15	0.098	[1]	fold change	1.47	0.14	[1]

Table caption. * Parameter could not be accurately determined in the respective study [1].

References

1. Ray AS, Murakami E, Basavapathruni A, Vaccaro JA, Ulrich D, et al. (2003) Probing the molecular mechanisms of AZT drug resistance mediated by HIV-1 reverse transcriptase using a transient kinetic analysis. Biochemistry 42: 8831–8841.
