# Supplementary material for: HIV-1 Polymerase Inhibition by Nucleoside Analogs: Cellular- and Kinetic Parameters of Efficacy, Susceptibility and Resistance Selection
Source: PLoS Comput Biol. 2012 Jan 19;8(1):e1002359. doi: 10.1371/journal.pcbi.1002359 (PMC3261923; doi:10.1371/journal.pcbi.1002359)
Supplement: Table S4 — Pre-steady state kinetic constants for nucleoside incorporation by human mitochondrial polymerase- . was set to value zero because of insufficient information. (PDF) [file pcbi.1002359.s004.pdf]

Table S4. Pre-steady state kinetic constants for nucleoside incorporation by human mitochondrial polymerase-γ

	K_D μM	k_{pol} [s^{-1}]	r_{exo} [s^{-1}]	ref.
dATP	0.8	45	-*	[1]
dTTP	0.6	25	-*	[1]
dCTP	0.8	37	-*	[1]
dGTP	0.9	43	-*	[1]
TFV-DP	40.3	0.21	0.0007	[2]
AZT-TP	187	0.2	0.0004	[2]
D4T-TP	0.045	0.24	0.0004	[2]
FTC-TP	62.9	0.0086	0.0048	[3, 4]
3TC-TP	9.2	0.125	0.015	[2]
CBV-TP	13	0.0018	0.0016	[2]

* r_{pyro} was set to value zero because of insufficient information.

References

1. Johnson AA, Johnson KA (2001) Fidelity of nucleotide incorporation by human mitochondrial DNA polymerase. J Biol Chem 276: 38090–38096.

2. Johnson AA, Ray AS, Hanes J, Suo Z, Colacino JM, et al. (2001) Toxicity of antiviral nucleoside analogs and the human mitochondrial DNA polymerase. J Biol Chem 276: 40847–40857.

3. Wendelsdorf KV, Song Z, Cao Y, Samuels DC (2009) An analysis of enzyme kinetics data for mitochondrial DNA strand termination by nucleoside reverse transcription inhibitors. PLoS Comput Biol 5: e1000261.

4. Honkoop P, Scholte HR, de Man RA, Schalm SW (1997) Mitochondrial injury. Lessons from the fialuridine trial. Drug Saf 17: 1–7.
