# Supplementary material for: HIV-1 Polymerase Inhibition by Nucleoside Analogs: Cellular- and Kinetic Parameters of Efficacy, Susceptibility and Resistance Selection
Source: PLoS Comput Biol. 2012 Jan 19;8(1):e1002359. doi: 10.1371/journal.pcbi.1002359 (PMC3261923; doi:10.1371/journal.pcbi.1002359)
Supplement: Text S1 — The supplementary text contains the modelling required to compute the probability to successfully complete reverse transcription (RT) in HIV-1, based on the parameters presented in the main manuscript. (PDF) [file pcbi.1002359.s005.pdf]

S1 Inhibition of Polymerization and Probability to Successfully Complete Reverse Transcription of HIV-1

The model presented in the main manuscript is built around the concept of time required for completion of the polymerization reaction. In particular, we derived a parameter $(1-\varepsilon)$, which denotes the residual polymerization in the presence of NAs. Here, we want to give an example of how inhibition of the polymerization reaction could alter the probability to successfully complete reverse transcription ρ_{RT}. The example will be based on HIV-1. For this particular virus the targeted process is reverse transcription, which takes place after the virus penetrated the target cell, but before it is stably integrated into the host cell's DNA [1], see also Fig. S1. If we assume that the decay kinetics of the HIV-RT complex is

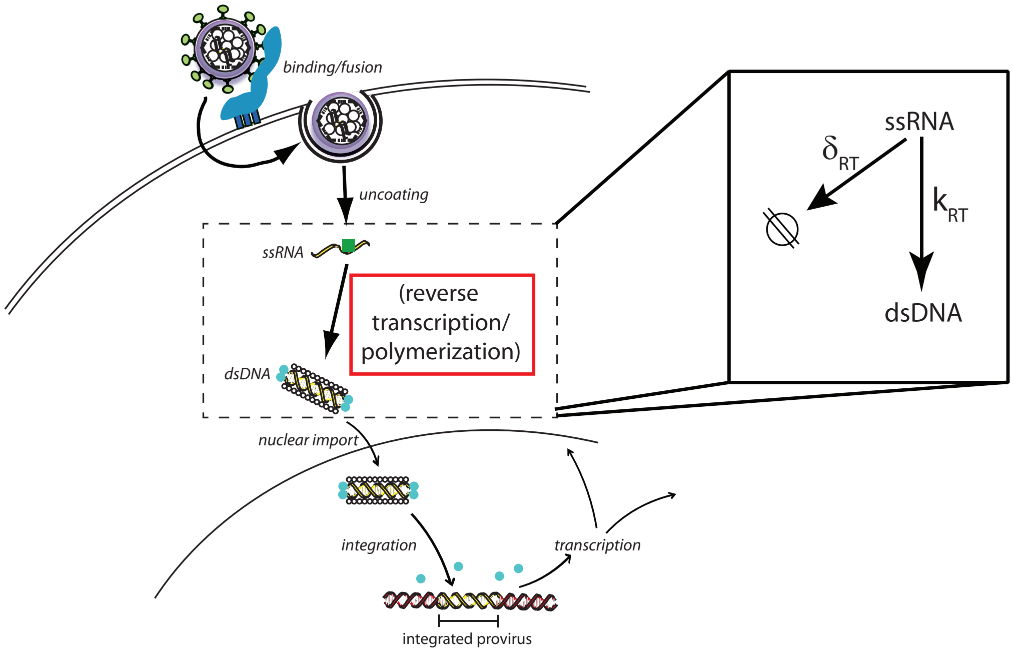

Figure S1. Reverse transcrition/polymerization during the life-cycle of HIV. The two competing processes of reverse transcription k_{RT} and the inactivation of the RT complex δ_{RT} are highlighted. Only if HIV succeeds to finalize RT, before its inactivation, subsequent steps of host cell infection can occur.

linear, then the probability to successfully finalize RT in the absence of treatment is given by

$$\rho_{\phi,\mathrm{RT}} = \frac{k_{\phi,\mathrm{RT}}}{k_{\phi,\mathrm{RT}} + \delta_{\mathrm{RT}}}, \tag{1}$$

where $k_{\phi,\mathrm{RT}}$ and δ_{RT} denote the rate of reverse transcription in the absence of drugs ϕ and the rate of decay of the RT complex [1/time]. Estimated values of $\rho_{\phi,\mathrm{RT}}$ range from 0.15 [2] to 0.5 [3] in unstimulated CD4$^+$ T-cells. Further, we assume that $k_{\phi,\mathrm{RT}} \approx k_{\phi,\mathrm{Pol}}$, namely that the sub-process of polymerization requires most time during reverse transcription in the absence of inhibitors (strand transfer, initiation and RNAse H, either occur in parallel or require a much smaller fraction of time compared to polymerization) [4]. Therefore, in

the presence of inhibitors we will also have $k_{\mathrm{NA,RT}} \approx k_{\mathrm{NA,Pol}}$, because NAs prolong the time required to finalize polymerization, i.e reduce k_{Pol}. It follows, that

$$\rho_{\mathrm{NA,RT}} = \frac{k_{\mathrm{NA,RT}}}{k_{\mathrm{NA,RT}}+\delta_{\mathrm{RT}}} \quad \text{(probability to finish RT in the presence of NAs),} \tag{2}$$

i.e. NAs decrease the probability that HIV can successfully finalize RT by decreasing k_{RT}. Therefore, NAs indirectly contribute to the clearance of HIV's RT-complex by intracellular inactivation processes. Using $k_{\mathrm{NA,Pol}} \approx (1-\varepsilon)\cdot k_{\phi,\mathrm{Pol}}$ and $k_{\phi,\mathrm{RT}} \approx k_{\phi,\mathrm{Pol}}$, we can finally set $k_{\mathrm{NA,RT}} \approx (1-\varepsilon)\cdot k_{\phi,\mathrm{RT}}$, where $(1-\varepsilon)$ denotes the residual polymerization, defined in eqs. (1-2) of the main article. After some rearrangement, we derive the relation of the residual probability to finalize RT $\frac{\rho_{\mathrm{NA,RT}}}{\rho_{\phi,\mathrm{RT}}}$ and residual polymerization $(1-\varepsilon)$:

$$\rho_{\mathrm{NA,RT}} \approx \frac{\rho_{\phi,\mathrm{RT}}}{\rho_{\phi,\mathrm{RT}}+\frac{1-\rho_{\phi,\mathrm{RT}}}{(1-\varepsilon)}} \tag{3}$$

$$\Rightarrow \frac{\rho_{\mathrm{NA,RT}}}{\rho_{\phi,\mathrm{RT}}} \approx \frac{1}{\rho_{\phi,\mathrm{RT}}+\frac{1-\rho_{\phi,\mathrm{RT}}}{(1-\varepsilon)}} \quad \text{(residual probability to finish RT),} \tag{4}$$

which is shown in Fig. S2 (left panel). In summary, it is possible to compute the residual probability to finalize RT $\frac{\rho_{\mathrm{NA,RT}}}{\rho_{\phi,\mathrm{RT}}}$ in the presence of NAs from the residual polymerization $(1-\varepsilon)$. This conversion does not affect the shape of the dose-response curve (Fig. S2, right panel), but results in a shift of the dose-response curve towards higher NA concentrations, i.e. polymerization is more strongly inhibited than the probability to finalize RT. The extend of the shift depends on the probability to finalize RT in the absence of inhibitors $\rho_{\phi,\mathrm{RT}}$. If RT is usually finalized in the absence of drugs, there will be a greater shift of the dose-response curve towards the right, i.e. the virus is less sensitive to treatment. Given the derivations

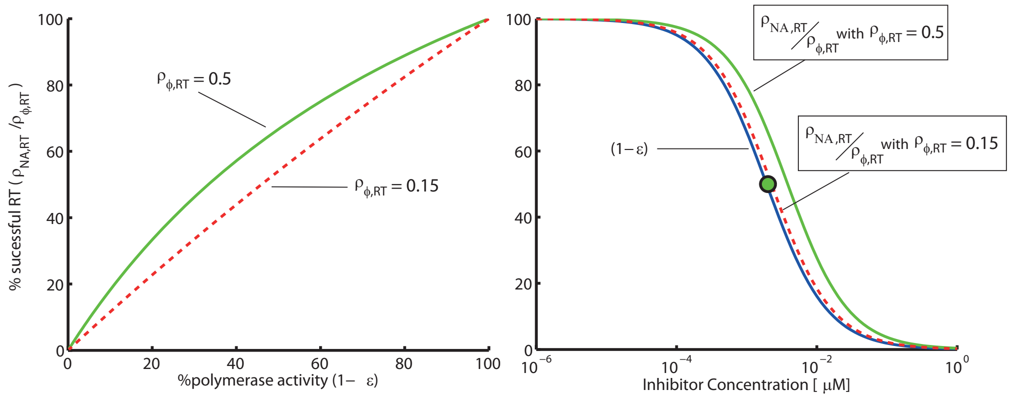

Figure S2. Relation of the residual probability to finalize RT and residual polymerization. Left: Relation of the residual probability to finalize RT $\frac{\rho_{\mathrm{NA,RT}}}{\rho_{\phi,\mathrm{RT}}}$ and residual polymerization $(1-\varepsilon)$ if the probability to finalize RT in the absence of inhibitors was $\rho_{\phi,\mathrm{RT}} = 15\%$ (dashed red line), or $\rho_{\phi,\mathrm{RT}} = 50\%$ (solid green line). Right: Comparison of the dose-response curve with regard to residual RNA-dependent polymerization in the presence of AZT-TP in resting $CD4^{+}$ T-cells (solid blue line) and the residual probability to finalize RT, if the probability to finalize RT in the absence of inhibitors was $\rho_{\phi,\mathrm{RT}} = 15\%$ (dashed red line), or $\rho_{\phi,\mathrm{RT}} = 50\%$ (solid green line).

and assumptions above, it is also possible to fully mechanistically model the effects of NAs (e.g. NRTIs), on the viral life cycle and virus load, using the equations provided in von Kleist et al. [1] (see eqs. (7)-(8) and supplementary eqs. (S21-S26) therein).

The equations above (in particular eq. (4)) also hold for other viruses, in which the processes of polymerization and inactivation are competing.

References

1. von Kleist M, Menz S, Huisinga W (2010) Drug-class specific impact of antivirals on the reproductive capacity of HIV. Plos Computational Biology 6: e1000720.

2. Pierson TC, Zhou Y, Kieffer TL, Ruff CT, Buck C, et al. (2002) Molecular characterization of preintegration latency in human immunodeficiency virus type 1 infection. J Virol 76: 8518–8531.

3. Zhou Y, Zhang H, Siliciano JD, Siliciano RF (2005) Kinetics of human immunodeficiency virus type 1 decay following entry into resting CD4+ T cells. J Virol 79: 2199–2210.

4. Thomas DC, Voronin YA, Nikolenko GN, Chen J, Hu WS, et al. (2007) Determination of the ex vivo rates of human immunodeficiency virus type 1 reverse transcription by using novel strand-specific amplification analysis. J Virol 81: 4798–4807.
